# Achieving high-sensitivity for clinical applications using augmented exome sequencing

**DOI:** 10.1186/s13073-015-0197-4

**Published:** 2015-07-16

**Authors:** Anil Patwardhan, Jason Harris, Nan Leng, Gabor Bartha, Deanna M. Church, Shujun Luo, Christian Haudenschild, Mark Pratt, Justin Zook, Marc Salit, Jeanie Tirch, Massimo Morra, Stephen Chervitz, Ming Li, Michael Clark, Sarah Garcia, Gemma Chandratillake, Scott Kirk, Euan Ashley, Michael Snyder, Russ Altman, Carlos Bustamante, Atul J. Butte, John West, Richard Chen

**Affiliations:** Personalis, Inc, 1330 O’Brien Drive, Menlo Park, California 94025 USA; Biosystems and Biomaterials Division, National Institute of Standards and Technology, Gaithersburg, Maryland USA; Institute for Computational Health Sciences, University of California, San Francisco, California 94158 USA; Departments of Bioengineering & Genetics, Stanford University, Stanford, California 94305 USA; Department of Genetics, Stanford University School of Medicine, Stanford, California 94305 USA; Center for Inherited Cardiovascular Disease, Stanford University School of Medicine, Stanford, California 94305 USA

## Abstract

**Background:**

Whole exome sequencing is increasingly used for the clinical evaluation of genetic disease, yet the variation of coverage and sensitivity over medically relevant parts of the genome remains poorly understood. Several sequencing-based assays continue to provide coverage that is inadequate for clinical assessment.

**Methods:**

Using sequence data obtained from the NA12878 reference sample and pre-defined lists of medically-relevant protein-coding and noncoding sequences, we compared the breadth and depth of coverage obtained among four commercial exome capture platforms and whole genome sequencing. In addition, we evaluated the performance of an augmented exome strategy, ACE, that extends coverage in medically relevant regions and enhances coverage in areas that are challenging to sequence. Leveraging reference call-sets, we also examined the effects of improved coverage on variant detection sensitivity.

**Results:**

We observed coverage shortfalls with each of the conventional exome-capture and whole-genome platforms across several medically interpretable genes. These gaps included areas of the genome required for reporting recently established secondary findings (ACMG) and known disease-associated loci. The augmented exome strategy recovered many of these gaps, resulting in improved coverage in these areas. At clinically-relevant coverage levels (100 % bases covered at ≥20×), ACE improved coverage among genes in the medically interpretable genome (>90 % covered relative to 10-78 % with other platforms), the set of ACMG secondary finding genes (91 % covered relative to 4-75 % with other platforms) and a subset of variants known to be associated with human disease (99 % covered relative to 52-95 % with other platforms). Improved coverage translated into improvements in sensitivity, with ACE variant detection sensitivities (>97.5 % SNVs, >92.5 % InDels) exceeding that observed with conventional whole-exome and whole-genome platforms.

**Conclusions:**

Clinicians should consider analytical performance when making clinical assessments, given that even a few missed variants can lead to reporting false negative results. An augmented exome strategy provides a level of coverage not achievable with other platforms, thus addressing concerns regarding the lack of sensitivity in clinically important regions. In clinical applications where comprehensive coverage of medically interpretable areas of the genome requires higher localized sequencing depth, an augmented exome approach offers both cost and performance advantages over other sequencing-based tests.

**Electronic supplementary material:**

The online version of this article (doi:10.1186/s13073-015-0197-4) contains supplementary material, which is available to authorized users.

## Background

Next-generation sequencing (NGS) technologies are increasingly used for the diagnosis of suspected genetic syndromes and cancer [1, 2]. Reductions in cost and time to diagnosis have made NGS-based testing a practical first-line tool in a diagnostic evaluation, potentially supplanting or supplementing other low-yield imaging, biochemical, histopathology, and genetic evaluations. Whole exome sequencing (WES) is a particularly efficient diagnostic method because it interrogates exome-wide variation in a single assay and can provide a genetic assessment even when candidate genes are not known, or when a disorder exhibits substantial phenotypic and genetic heterogeneity. Several studies [2–7] have demonstrated the ability of WES to reveal medically significant variants, even in cases where prior diagnostic tests were performed.

Sequencing-based diagnostic tests require adequate breadth and depth of coverage to ensure high clinical sensitivity. Despite the rapid adoption of WES technologies in clinical decision-making, the extent and consistency of coverage over medically relevant variants is poorly understood. Single-gene and gene-panel tests are often evaluated using patient-derived samples that harbor known disease-related mutations. In contrast, it is not feasible to evaluate accuracy among all potential variants associated with all known diseases captured on an exome-wide or genome-wide basis. However, recent guidance on the evaluation of NGS technologies for use in clinical settings [8–10] establishes criteria for evaluating the accuracy of variant detection in WES. As recently demonstrated [11–13], this includes the calculation of false positive (FP) and false negative (FN) rates using well-characterized reference materials (RM) and the reporting of depth of coverage and breadth of coverage statistics.

Using these guidelines, we examine the coverage and accuracy obtained with currently available exome capture technologies and whole genome sequencing (WGS). With a pre-defined list of medically-relevant protein-coding and non-coding sequences, we identify regions of the genome that are poorly covered and inaccurately identified with these technologies. Finally, we present an Accuracy and Content Enhanced (ACE) augmented exome strategy that improves coverage in these regions and provides variant detection sensitivities not typically achieved with other commercially available exome platforms.

## Methods

### Samples and sequencing

Exome capture was performed using the well-characterized cell-line sample, NA12878 [14], a prospective RM at the time of this study [15], using two recently developed commercial WES capture kits: Agilent SureSelect Human All Exon v5 plus untranslated regions (UTR) (SS) and Agilent SureSelect Clinical Research Exome (SSCR) according to manufacturers’ recommendations. Manufacturer protocols were modified to adjust the average library insert length to approximately 250 bp and included the use of KAPA HiFi DNA Polymerase (Kapa Biosystems) instead of Herculase II DNA polymerase (Agilent), given recent evidence of improved on-target capture performance with high-fidelity polymerases [16]. Sequencing was performed using HiSeq 2500 (Illumina, San Diego, CA, USA) sequencers with single lane, paired-end 2 × 101 bp reads and Illumina’s proprietary Reversible Terminator Chemistry (v3). In addition, raw read-data files (FASTQ 2 × 101 bp reads) using the NimbleGen SeqCap EZ Human Exome Library v3.0 (NG) exome capture kit [17] and lllumina’s Nextera Rapid Capture Exome (NX) [18] were obtained from the sequence read archive (SRA) under accession SRX731649 [11] and from Illumina’s BaseSpace repository [19], respectively. For NG and NX, reads were combined across replicate runs of the same sample (NA12878) in order to obtain the coverage depth needed for subsequent analysis. For ACE, target probes were prepared to enhance coverage within sets of biomedically and medically relevant genes as described in additional materials (Additional files 1 and 2). Details regarding ACE assay robustness and reproducibility are described in Additional file 1.

Preserving read pair information, the original amount of sequence data collected for each WES platform was randomly downsampled to control either the total amount of sequence data in Gigabases (Gb) or the mean depth of coverage in each platform’s target regions. Downsampling to a fixed amount of sequence data has the advantage of controlling for the combination of breadth (footprint) and depth of sequencing - two parameters that are key determinants of WES assay performance. Total sequence data can also be more easily related to overall-sequencing costs given that the target regions (and mean coverage within target regions) vary widely among WES platforms. Conversely, 100× average depth of coverage is commonly referred to as the minimum amount of coverage needed in clinical applications, regardless of the total amount of sequence data obtained.

100× mean coverage depth within each platform’s target region was obtained using the following amounts of sequence data: 13.8 Gb (SS), 8.9 Gb (SSCR), 18.6 Gb (NX), 13.4 Gb (NG), and 13.8 Gb (ACE). In addition, 12 Gb of sequence data were obtained for each WES and ACE platform resulting in mean coverages of 88.3× (SS), 132.2× (SSCR), 91.1× (NX), 91.9 (NG), and 86.9× (ACE) in the respective target regions. Using a standard Illumina TruSeq PCR-free protocol, we also obtained 100.0 Gb WGS data resulting in a mean coverage depth of 31.5×. FASTQ files resulting from the downsampled data used in this study or 31.5× WGS are available from SRA under accession PRJNA289286.

### Alignment, mapping, and variant identification

For all platforms, raw sequence data were in FASTQ format and were analyzed with standard Phred-scale quality scores. Gapped alignment was performed using the Burrows-Wheeler Aligner (v.0.6.2) [20] combined with Picard (v.1.74) [21] and the Genome Analysis Toolkit (GATK v3.1) [22] base quality score recalibration to perform sequence alignment and base quality scoring. Data were aligned to the hs37d5 genome [23], producing compressed Binary Alignment Map format files. GATK’s Unified Genotyper module provided the core set of SNV and InDel calls and quality metrics using both GATK’s variant quality score recalibration (VQSR) (for SNVs) and hard-filtering (for InDels), per GATK best practices documentation [24]. SNV and small InDels were reported in variant call format (VCF).

### Coverage and accuracy statistics

For each platform, the mean coverage depth over each exon was calculated from the base-resolved coverage depth integrated over the exon length, considering only aligned bases with high-quality mapping (Q ≥20) and base-quality (Q ≥20) scores. Gene-specific mean coverages were calculated as the mean coverage of the constitutive exons weighted by each exon length. We also report the percent of exonic bases reaching a minimum coverage threshold of ≥20×, a level of coverage depth necessary to call heterozygous SNVs with approximately 99 % sensitivity in WES and WGS data [25, 26]. Using a stringent definition of high-quality coverage, we termed a gene ‘finished’ when 100.0 % of its exonic bases met this threshold.

To evaluate relative platform performance, we calculated coverage and accuracy statistics for ACE and other commercially available platforms within commonly-defined medically relevant target regions. Accuracy was assessed by utilizing two reference ‘gold standard’ call-sets available for the NA12878 RM from the National Institutes of Standards and Technology (NIST) Genome in a Bottle (GIB) consortium. Briefly, the NIST-GIB high-confidence call-set (GIBv2.18, 16 December 2013) [27] is restricted to high-confidence regions of the genome based on arbitration of SNV, InDel, and homozygous reference genotype calls among multiple sequencing platforms, aligners, and variant callers. It further filters locations in an effort to remove regions of the genome where the likelihood of an incorrect genotype call is increased. A second call-set was used that contains variants with evidence from >1 platform but may fail published arbitration rules [27] or fall into regions that are difficult to sequence. Despite a higher likelihood of benchmark-set errors in these regions, this second ‘less restrictive’ call-set is useful in evaluating the relative sensitivity to variants in known problematic regions (for example, areas of high GC) that are typically excluded from high-confidence call-sets and exome-based target regions.

Sequencer, alignment, and variant calling parameters were set to be identical in the analysis of all exome-based sequencing platforms (WES and ACE) with the exception of the target capture region used, which is specific to each platform. Error rates were derived from the comparison of observed variant call-sets to reference call-sets within the medically interpretable genome (MIG), within a target region common (that is, the overlap/intersection) to all exome-based platforms (Common Target File), within a subset of predicted moderate-high impact variants occurring in any of the platform-specific target files (Union Target File), and within regions of >70 % GC content. True positive (TP) observed calls matched the reference call in position, genotype and alternate bases, and were based on those variants that are callable (that is, the proportion of variants that are detected at or above the predefined alignment, mapping quality and variant calling quality thresholds). FP and FN rates were calculated based on the use of GATK’s VQSR module derived VQSLOD score (log odds (variant / no variant) cutoffs for SNVs. A set of hard-filter thresholds, which includes the Phred-scaled quality scores (QUAL, −10log_10_ P(variant / no variant)), were used for evaluating InDels. These cutoffs discretized the variant call likelihood scores into a series of categorical ‘FILTER’ levels. The PASS level was used as a threshold for both variant types across all platforms, which is estimated to capture 99.5 % of known TP SNVs [24]. Both genotyping and mischaracterization errors were included as FP and FN errors. 95 % confidence intervals for sensitivity and the false discovery rate (FDR) were based on an exact binomial test [28]. Pair-wise comparisons of observed sensitivities across platforms was done using *X*^2^(chi-square, df = 1), with a significant level of α = 0.01.

### Establishing the medically interpretable genome

**Fig. 1:**
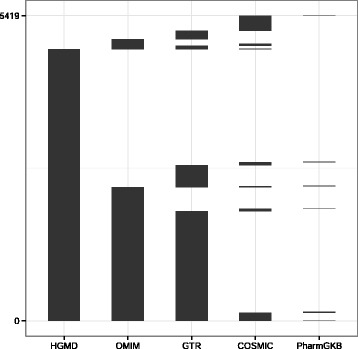
A total of 5,419 genes in the MIG drawn from five data sources. The bulk (98 %) of genes came from HGMD, OMIM, and GTR with additional genes supplemented from COSMIC (67) and PharmGKB (1). Areas of vertical overlap indicate genes common across multiple sources

We first assembled a list of 5,419 unique genes in which mutations have been causally implicated in disease or disease-related drug response. This list included genes that: (1) are part of an existing clinical test; (2) are documented in published literature as pharmacogenes; or (3) have a causal association with Mendelian disease, inherited disease, or cancer. This literature-based gene set was constructed by combining three public data-sources: a subset of Mendelian Disease genes catalogued in Online Mendelian Inheritance in Man [29] (OMIM), the Human Gene Mutation Database [30] (HGMD, v2013.4), and clinical genetic tests submitted to the Genetic Testing Registry (GTR, 07/14 data release) [31]. This list was then supplemented with genes drawn from the Cancer Gene Census (COSMIC, 7/14 data release) [32], and a subset of PharmGKB (04/14 data release) [33], which included genes classified in the Very Important Pharmacogenes (VIP) project and/or those with dosing guidelines available in the Clinical Pharmacogenetics Implementation Consortium (CPIC). Figure 1 identifies the number of genes drawn from these five sources.

Gene redundancies, due to the use of gene synonyms in source databases, were eliminated by mapping gene symbols to the currently approved HGNC and NCBI identifiers. Chromosomal location information for each gene was based on NCBI annotation (release 105), whereby regions were defined by collapsing all annotated transcripts per region. Collectively, the genomic regions defined by these genes and the reference transcripts are referred to as the ‘medically interpretable genome’ (MIG) (Additional file 3). Notably, the MIG contains 97 % of the genes defined by the International Collaboration for Clinical Genomics (ICCG) consortium as belonging to the ‘medical exome’, after filtering the ICCG set to remove redundant genes and unmappable gene locations. The MIG incorporates an additional 1,281 genes not found in the ICCG set. Since a female derived sample (NA12878) was used in this study, 20 genes occurring on the Y chromosome were excluded from the MIG for subsequent analysis.

## Results

### Coverage in the MIG

**Fig. 2:**
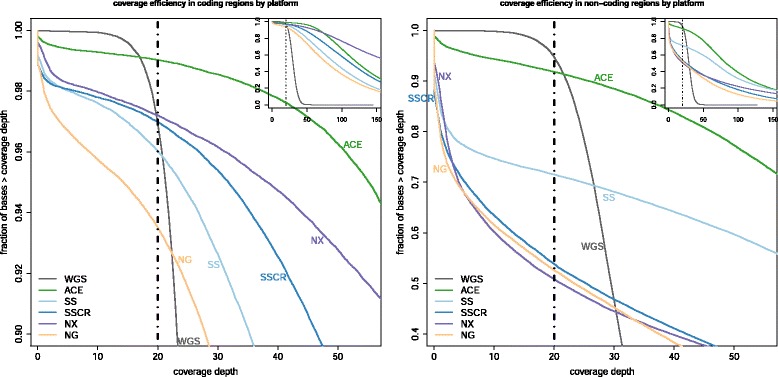
Coverage efficiency in the medically interpretable genome (MIG). Shown is the cumulative distribution of on-target sequence coverage obtained from sequencing NA12878 across multiple platforms: Personalis Accuracy and Content Enhanced (ACE) Clinical Exome, Agilent SureSelect Clinical Research Exome (SSCR), Agilent SureSelect Human All Exon v5 plus untranslated regions (UTR) (SS), lllumina’s Nextera Exome Enrichment (NX), NimbleGen SeqCap EZ Human Exome Library v3.0 (NG), and 31× whole-genome sequencing (WGS) using an Illumina PCR-free protocol. For clinical applications, we indicate ≥20× as the minimum coverage threshold required (gray line) among all coding (left) and non-coding (right) regions. For reference, insets show an expanded distribution of sequence coverage. ACE and conventional WES data are normalized to 100× mean target coverage

We compared coverage performance among ACE, four conventional WES platforms (SS, SSCR, NX, NG) and WGS using the DNA from NA12878. WES and ACE platforms were compared after normalizing to both 12 Gb of total sequence data and to 100× mean coverage depth in each platform’s respective target regions. At 100× mean-target coverage (ACE, WES) and 31.5× (100 Gb) WGS, the mean coverage depth observed in the MIG was: 102.7× (SS), 125.1× (SSCR), 208.8× (NX), 95.5× (NG), 138.0× (ACE), and 29.5× (WGS). The coverage efficiency observed within MIG genes across all platforms when normalized for 100× mean target coverage depth is shown in Fig. 2. The distribution of base-quality reads observed at different levels of coverage depths is shown, centered at a clinically relevant minimum coverage of ≥20× (vertical gray line). At ≥20×, ACE covers >99 % of bases in protein coding regions and 93 % of bases in the non-coding regions compared to 93-97 % of protein coding and 50 %-73 % non-coding bases covered across WES platforms. WGS covered 97 % and 95 % of all bases in coding and non-coding regions respectively (Fig. 2). Notably, low-coverage in non-coding regions of the genome is expected with SSCR, NX, and NG, which do not substantially include non-coding areas (for example, UTRs) in the target design.

**Fig. 3:**
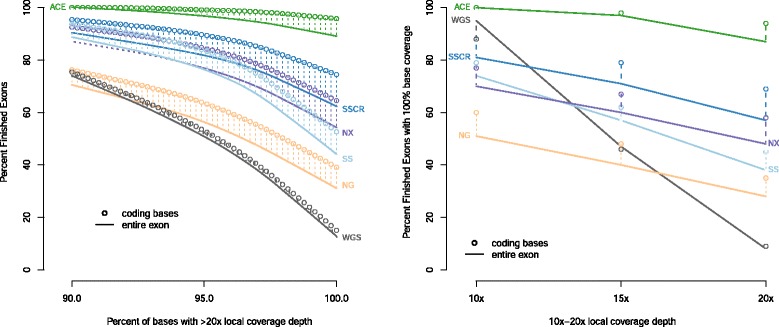
Relationship between the percentages of MIG exons ‘finished’ as the coverage stringency varies. The left graph shows the percentage of MIG exons (y-axis) with ≥90.0-100.0 % of bases covered at ≥20× depth (x-axis) among different platforms using data obtained on NA12878. The right graph shows the percentage of finished exons (y-axis) with 100.0 % base coverage as the local coverage depth varies ≥10-20× (x-axis). At higher coverage stringencies, ACE finishes more exons than other WGS or WES assays in regions defined as the entire exon (solid curves) or only the subset of coding-regions (circles). ACE and conventional WES data are normalized to 100× mean target coverage

We next examined the percentage of MIG genes ‘finished’ as the criterion for base coverage varied. Figure 3 shows the number of finished MIG genes observed in NA12878 with ≥90.0-100.0 % of constituent exonic bases covered at ≥20×. ACE achieved 100.0 % base coverage at ≥20× in approximately 90 % of the MIG genes. Conventional WES platforms (SS, SSCR, NX, NG) finished 30-65 % of genes at this level whereas WGS finished 10 %. If the stringency for per-gene percent coverage is reduced to ≥90.0 % of exonic bases, 100 % of genes are finished at ≥20× with ACE; between 65 % and 90 % of genes are finished among WES; and 75 % of genes are finished with WGS. Conversely, we also examined the percentage of finished MIG genes as the coverage depth was in the range of ≥10-20× (Fig. 2, right). Generally, at lower minimum coverage levels (that is, 10×) ACE finished the most genes (100 %) followed by WGS (96 %), SSCR (81 %), SS (75 %), NX (70 %), and NG (51 %). Relative WES platform performance remained consistent as the coverage finishing threshold increased to ≥20×, with ACE continuing to cover a higher percentage of bases at higher depths. In contrast, WGS coverage performance decreased sharply as coverage stringency increased, finishing only 10 % of genes at ≥20 × .

**Fig. 4:**
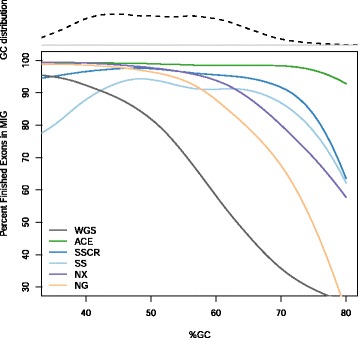
Relationship between GC content and the percentages of MIG exons ‘finished’ by platform. Regions with >30-80 % GC content (x-axis) represent 99 % of exons in the MIG. Finishing is determined by 100 % base coverage at ≥20×

The relative breadth and depth of coverage across exons with varying GC composition was similar to the relative platform performance observed in the MIG set. ACE finished a larger percentage of MIG exons compared to other WES and WGS platforms (Fig. 4), finishing >90 % of exons regardless of the amount of GC content. Other platforms showed a decline in the number of finished exons as the percentage of GC increased, with some platforms (WGS, NG, NX) showing substantial reductions at >50 % GC content.

Analyses were repeated after re-normalizing WES and ACE data to 12 Gb of total sequence data (Additional file 4). Relative performance among platforms was consistent with the results reported above, which are based on data normalized to 100× mean coverage within each platforms target region. For reference, a summary of platform parameters and sequencing statistics is shown in Additional file 5.

### Coverage performance in the ACMG genes and known disease-associated variants

Included within the MIG gene set are 56 genes that per ACMG guidelines [34] are recommended for examination and reporting of secondary findings during clinical genomic testing. Although concerns over the accuracy of sequencing platforms in clinically relevant regions of the genome have been widely discussed [8, 35], the lack of sensitivity of WES and WGS to known variants occurring in genes of the ACMG secondary findings list have highlighted the extent of these inaccuracies [36, 37]. The coverage of these genes and their constituent variants by these platforms illustrates how variations in design can impact clinical decision making, presuming that a lack of sensitivity to variants within these genes: (1) affects the reporting of secondary findings; and (2) is representative of other pathogenic variants not specifically assessed in this study.

Using WES and ACE data normalized to 100× coverage depth, the per-gene mean coverage observed among the 56 genes was in the range of 41-371× for WES, 24-36× for WGS, and 92-234× for ACE (Additional file 6). Ten (18 %) of the 56 genes failed to reach our predefined level of coverage (100 % bases covered at ≥20×) in any of the conventional WES platforms (SS, SSCR, NG, NX). Among these genes, eight had some proportion of their exonic bases covered at a higher depth (that is, covered at ≥20×) with ACE (*MEN1, RB1, TGFBR1, PKP2, KCNQ1, KCNH2, PCSK9, RYR1*) and two showed improved coverage with WGS (*MEN1, TGFBR1*). Exome-based platforms (WES, ACE) generally showed substantially improved breadth and depth of coverage compared to 31× WGS for these 56 genes. Fifty-four genes had some proportion of their constituent bases inadequately covered (<20×) with 31× WGS. Of these, 53 genes had a larger fraction of exonic bases covered at ≥20× using ACE and 52 had a larger fraction covered with at least one of the conventional WES platforms (SS, SSCR, NX, NG). Two genes with some proportion of their exonic bases inadequately covered (<20×) with ACE had these bases covered to ≥20× by NX (*PMS2*) or WGS (*MEN1*). The individual platform rankings based on the number of genes with 100 % base coverage at ≥20×, were ACE (51 genes) > SSCR (39 genes) > NX (36 genes) > SS (15 genes) > NG (12 genes) > and WGS (2 genes) (Additional file 6).

**Fig. 5:**
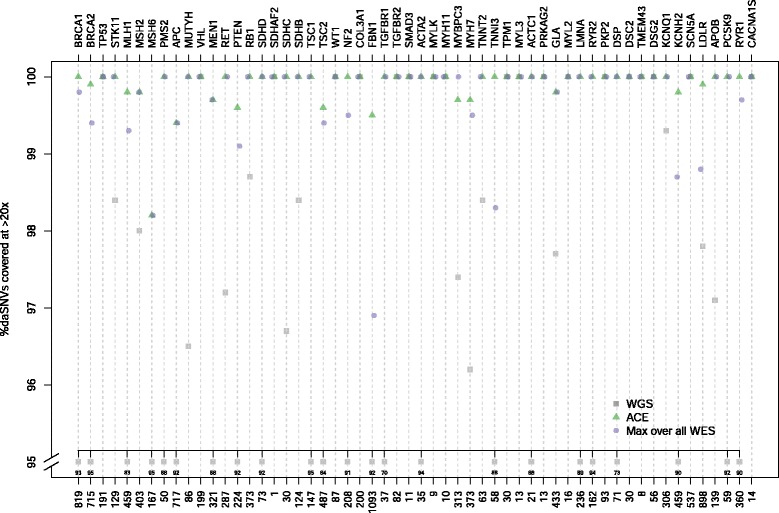
Disease-associated variants covered at ≥20× for 56 genes in the ACMG gene list. The x-axis labels indicate the total number of disease-associated SNVs (daSNVs) drawn from HGMD for each ACMG gene; and the y-axis indicates the percentage of those variants covered at ≥20×. For brevity, only the highest obtained percentage (Max over all WES) observed across all conventional WES (SS, SSCR, NX, NG) platforms is shown. Seventeen of the 56 genes failed to have some fraction of their daSNVs covered at ≥20× among any of the conventional WES platforms. On a gene basis, the platforms with the highest to lowest number of genes with constituent daSNVs adequately covered included ACE (51 genes with 100 % daSNVs covered at ≥20×), SSCR (39 genes), NX (36 genes), SS (15 genes), NG (12 genes), and WGS (2 genes). The y-axis is truncated at 95 %, with truncated points labelled accordingly

Several regions inadequately covered by WES platforms encompass disease-associated variants. Using 12,535 documented disease-associated SNVs (daSNV) in HGMD (version 2013_01) for the 56 ACMG genes as a ‘truth’ set, we extended our analysis to examine the fraction of daSNV loci covered at ≥10-25× with WES, ACE, and WGS platforms. Figure 5 shows the percentage daSNVs covered at ≥20× with more extensive tabular results (≥10×, ≥15×, ≥20×, ≥25×) reported in Additional file 7. For brevity, only the highest obtained base coverages achieved (Max) across all WES platforms (SS, SSCR, NX, NG) are shown. Depending on the platform used, 0.8-9.6 % (96–1,200 loci) of the daSNVs showed inadequate coverage (<20×) with conventional WES compared to 6.0 % (756 loci) for WGS and 0.2 % (26 loci) for ACE. Coverage shortfalls were spread across 41 genes, with 2,134 (17 %) daSNVs showing <20× coverage in at least one platform (WES, ACE, or WGS) (Additional file 8). Among these loci, the platforms with the highest to lowest number of loci with adequate coverage depth (≥20×) were: ACE (1,836 daSNVs), SSCR (1,727), NX (1,653), SS (1,435), NG (1,100), and WGS (968).

Relative gene and daSNV coverage performance between platforms and the differences observed between platforms were consistent regardless of the normalization scheme used (total sequence data or mean coverage) for exome-based data. For reference, results using each method are reported alongside each other in additional materials (Additional files 6, 7, and 8).

### Accuracy and characteristics of detected variants

Inadequate coverage, together with errors occurring in downstream alignment and variant calling, reduces the ability to accurately identify and characterize variants. Since ACE extends coverage of conventional WES to include all medically interpretable regions of the genome and targets genomic areas that are challenging to sequence, we quantified its impact on the accuracy of variant calls in: (1) the MIG; (2) genomic regions that are overlapping among exome-based (that is, ACE, WES) platforms (Common Target File); (3) functionally impactful genomic regions targeted among any exome-based platforms (Union Target File); and (4) areas of high GC content. The Common Target File allowed us to evaluate relative variant sensitivity without regard to platform-specific target design. Differences among platforms would presumably be based on variations in depth of coverage and coverage efficiency rather than due to the selective exclusion of some regions by specific capture kits (for example, the exclusion of UTRs by SSCR, NX, NG). In contrast, the Union Target File allowed us to evaluate how differences in each platforms target region (for example, differences in targeted non-coding and coding regions) impacted accuracy among variants with putative functional impact. Loci within platform specific target files were annotated with information about genomic location (for example, intron, exon, intergenic, intragenic, coding region) and predicted deleterious impact (for example, low, moderate, high, modifier/other) [38]. Regions containing loci within high (frame-shift, stop-gain, splice-site acceptor, splite-site donor, start lost, stop lost) and moderate (non-synonymous coding, codon change plus deletion/insertion, codon deletion/insertion) impact regions were combined into the Union Target File. Non-synonymous coding mutations contributed most (99 %) to the moderate-impact class in the Union Target File whereas 60 % of high-impact variants were splice-site donor/acceptor loci, followed by frame-shift mutations (20 %), stop-gain (12 %), and start/stop-lost (8 %).

For each platform, error rates and accuracy are presented in terms of the interval tested, which consists of high-confident variant loci within the MIG (Table 1, left); Common Target File (Table 1, middle); and Union Target File (Table 1, right) or a less-restrictive set of loci within subsets of GC-rich regions (Table 2). For reference, the set of genomic regions comprising the Common Target File and Union Target File and a catalogue of all 792,245 exonic regions with >70 % GC content among 20,000 genes are provided (Additional files 9, 10, and 11). Information about resources used in constructing reference and target regions is included in Additional file 12.

**Table 1:** Accuracy across target regions. Errors, Sensitivity, and FDR for the ACE, WGS, SSCR, SS, NX, and NG platforms based on evaluation of observed variant calls using data normalized to 100× mean coverage (conventional WES and ACE) or 31× WGS. Calculations are based on position and genotype matching to the GIBv2.18 high-confident call-set within the MIG (left), a target region common to all ACE and WES platforms (middle, Common Target File), and a target region aggregated across all ACE and WES specific target files that contain moderate-impact and high-impact loci (right, Union Target File)

		MIG					Common Target File					Union Target File				
		TP	FP	FN	%Sens	%FDR^a^	TP	FP	FN	%Sens	%FDR^a^	TP	FP	FN	%Sens	%FDR^a^
					95%CI	95%CI				95%CI	95%CI				95%CI	95 % CI
ACE	SNV	5362	5	62	98.9	0.1	7133	12	90	98.8	0.2	7486	6	191	97.5	0.1
					98.5-99.1	<0.1-0.2				98.5-99.0	0.1-0.3				(97.1-97.8)	(<0.1-0.2)
	InDel	34	1	2	94.4	2.9	83	0	0	100	<0.1	198	3	16	92.5	1.5
					81.3-99.3	0.1-14.9				95.7-100	<0.1-4.3				(88.1-95.7)	(0.3-4.3)
WGS^b^	SNV	5309	2	115	97.9	<0.1	7076	6	147	98.0	0.1	7479	2	198	97.4	<0.1
					97.5-98.2	<0.1-0.1				97.6-98.3	<0.1-0.2				(97–97.8)	(<0.1-0.1)
	InDel	33	1	3	91.7	2.9	78	0	5	94.0	<0.1	197	2	17	92.1	1.0
					77.5-98.2	0.1-15.3				86.5-98.0	<0.1-4.6				(87.6-95.3)	(0.1-3.6)
SSCR	SNV	5341	4	83	98.5	0.1	7107	11	116	98.4	0.2	7443	4	234	97.0	0.1
					98.1-98.8	<0.1-0.2				98.1-98.7	0.1-0.3				(96.5-97.3)	(<0.1-0.1)
	InDel	34	2	2	94.4	5.6	82	0	1	98.8	<0.1	194	4	20	90.7	2
					81.3-99.3	0.7-18.7				93.5-100	<0.1-4.4				(85.9-94.2)	(0.6-5.1)
SS	SNV	5355	2	69	98.7	<0.1	7126	5	97	98.7	0.1	7468	3	209	97.3	<0.1
					98.4-99.0	<0.1-0.1				98.4-98.9	<0.1-0.2				(96.9-97.6)	(<0.1-0.1)
	InDel	33	2	3	91.7	5.7	82	0	1	98.8	<0.1	192	5	22	89.7	2.5
					77.5-98.2	0.7-19.2				93.5-100	<0.1-4.4				(84.8-93.4)	(0.8-5.8)
NX	SNV	5240	4	184	96.6	0.1	7020	8	203	97.2	0.1	6754	10	923	88.0	0.1
					96.1-97.1	<0.1-0.2				96.8-97.6	<0.1-0.2				(87.2-88.7)	(0.1-0.3)
	InDel	33	2	3	91.7	5.7	77	2	6	92.8	2.5	160	6	54	74.8	3.6
					77.5-98.2	0.7-19.2				84.9-97.3	0.3-8.8				(68.4-80.4)	(1.3-7.7)
NG	SNV	5190	31	234	95.7	0.6	6900	39	323	95.5	0.6	7065	38	612	92.0	0.5
					95.1-96.2	0.4-0.8				95.0-96.0	0.4-0.8				(91.4-92.6)	(0.4-0.7)
	InDel	31	4	5	86.1	11.4	74	2	9	89.2	2.6	168	10	46	78.5	5.6
					70.5-95.3	3.2-26.7				80.4-94.9	0.3-9.2				(72.4-83.8)	(2.7-10.1)

*FDR* false discovery rate, *FN* false negatives, *FP* false positives, *MIG* medically interpretable genome, *SENS* Sensitivity, *TP* true positives

^a^FDR is used in lieu of specificity due to a large skew in the TN, FP class distribution

^b^In WGS data, there was no difference in error rates when using either VQSLOD scores or hard-thresholding cutoffs for InDels

**Table 2:** Accuracy in high-GC rich regions. Errors, Sensitivity, and FDR for the ACE, WGS, SSCR, SS, NX, and NG platforms based on evaluation of observed variant calls using data normalized to 100× mean coverage (conventional WES and ACE) or 31× WGS. Calculations are based on position and genotype matching to the GIBv2.18 less restrictive call-set within the MIG (left), a target region common to all ACE and WES platforms (middle, Common Target File), and a target region aggregated across all ACE and WES specific target files that contain moderate-impact and high-impact loci (right, Union Target File)

		MIG					Common Target File					Union Target File				
		TP	FP	FN	%Sens	%FDR^a^	TP	FP	FN	%Sens	%FDR^a^	TP	FP	FN	%Sens	%FDR^a^
					95%CI	95%CI				95%CI	95%CI				95%CI	95 % CI
ACE	SNV	518	0	16	97.0	<0.1	706	1	22	97.0	0.1	562	2	30	94.9	0.4
					95.2-98.3	<0.1-0.7				95.5-98.1	<0.1-0.8				(92.8-96.6)	(<0.1-1.3)
	InDel	18	1	1	94.7	5.3	23	0	0	100	<0.1	37	0	3	92.5	<0.1
					74.0-99.9	0.1-26.0				85.2-100	<0.1-14.8				(79.6-98.4)	(<0.1-9.5)
WGS^b^	SNV	499	0	35	93.4	<0.1	701	0	27	96.3	<0.1	573	0	19	96.8	<0.1
					91.0-95.4	<0.1-0.7				94.6-97.5	<0.1-0.5				(95.0-98.1)	(0–0.6)
	InDel	18	0	1	94.7	<0.1	23	0	0	100	<0.1	38	0	2	95.0	<0.1
					74.0-99.9	<0.1-18.5				85.2-100	<0.1-14.8				(83.1-99.4)	(<0.1-9.3)
SSCR	SNV	504	1	30	94.4	0.2	684	4	44	94.0	0.6	545	2	47	92.1	0.4
					92.1-96.2	<0.1-1.1				92.0-95.6	0.2-1.5				(89.6-94.1)	(<0.1-1.3)
	InDel	17	1	2	89.5	5.6	21	1	2	91.3	4.5	37	0	3	92.5	<0.1
					66.9-98.7	0.1-27.3				72.0-98.9	0.1-22.8				(79.6-98.4)	(<0.1-9.5)
SS	SNV	497	2	37	93.1	0.4	704	0	24	96.7	<0.1	562	1	30	94.9	0.2
					90.6-95.1	<0.1-1.4				95.1-97.9	<0.1-0.5				(92.8-96.6)	(<0.1-1)
	InDel	16	2	3	84.2	11.1	21	0	2	91.3	<0.1	37	0	3	92.5	<0.1
					60.4-96.6	1.4-34.7				72.0-98.9	<0.1-16.1				(79.6-98.4)	(<0.1-9.5)
NX	SNV	465	1	69	87.1	0.2	650	1	78	89.3	0.2	484	0	108	81.8	<0.1
					83.9-89.8	<0.1-1.2				86.8-91.4	<0.1-0.9				(78.4-84.8)	(<0.1-0.8)
	InDel	19	0	0	100	<0.1	21	0	2	91.3	<0.1	31	1	9	77.5	3.1
					82.4-100	<0.1-17.6				72.0-98.9	<0.1-16.1				(61.5-89.2)	(0.1-16.2)
NG	SNV	346	6	188	64.8	1.7	436	14	292	59.9	3.1	373	10	219	63.0	2.6
					60.6-68.8	0.6-3.7				56.2-63.5	1.7-5.2				(59.0-66.9)	(1.3-4.7)
	InDel	11	0	8	57.9	<0.1	11	1	12	47.8	8.3	20	1	20	50.0	4.8
					33.5-79.7	<0.1-28.5				26.8-69.4	0.2-38.5				(33.8-66.2)	(0.1-23.8)

*FDR* false discovery rate, *FN* false negatives, *FP* false positives, *MIG* medically interpretable genome, *SENS* Sensitivity, *TP* true positives ^a^FDR is used in lieu of specificity due to a large skew in the TN, FP class distribution.

^a^FDR is used in lieu of specificity due to a large skew in the TN, FP class distribution

^b^In WGS data, there was no difference in error rates when using either VQSLOD scores or hard-thresholding cutoffs for InDels

Using WES and ACE data normalized to 100× mean coverage depth, sensitivities across intervals ranged from 88-99 % for SNVs and 75-100 % for InDels. ACE yielded the highest sensitivities (>97.5 % SNVs; >92.5 % InDels) relative to other platforms across all intervals (Table 1). Based on sensitivities to SNVs and InDels, the relative rank of platform performance in the MIG and Common Target File were similar: ACE > SS > SSCR > WGS > NX > NG; whereas the relative rank of platform performance in the Union Target File was ACE > WGS > SS > SSCR > NG > NX. FDRs for SNVs were low across all platforms (<1 %) regardless of the interval used. For InDels, the FDR was generally highest among NG and NX across intervals. The use of the VQSLOD score for InDels, as is sometimes recommended given the larger amount of data available from WGS [24], had no effect on InDel specific errors. Regardless of the interval used, observed differences in SNV sensitivities were small across platforms. ACE showed significantly (*P* <0.01) improved sensitivity for SNVs compared to NX and NG and in some cases WGS (MIG: ACE vs. WGS *X*^2^ = 16.1, *P* <0.01; ACE vs. NX *X*^2^ = 61.9, *P* <0.01; ACE vs. NG *X*^2^ = 102.7, *P* <0.01; Common Target File: ACE vs. WGS *X*^2^ = 13.9, *P* <0.01; ACE vs. NX *X*^2^ = 44.5, *P* <0.01; ACE vs. NG *X*^2^ = 135.3, *P* <0.01; Union Target File: ACE vs. WGS *X*^2^ = 0.1, *P* = 0.72; ACE vs. NX *X*^2^ = 518.6, *P* <0.01; ACE vs. NG *X*^2^ = 232.9, *P* <0.01); whereas no statistical significant improvement in SNV sensitivity was observed with ACE compared to SS or SSCR.

Increased breadth or depth of coverage is only asymptotically related to a higher capture efficiency, partly due to biases that occur with high-GC content [26]. These highly variable regions produce ‘gaps’ with levels of coverage insufficient for resolving disease causing variants [39]. Given the improved coverage characteristics of ACE in high GC content areas (Fig. 4), we examined its impact on accuracy in GC-rich regions. In the subset of the MIG and Common Target File containing >70 % GC content, ACE generally outperformed other platforms (Table 2) based sensitivities to SNVs (97.0 %) and InDels (>94.7 %). With the exception of NG and NX, however, the differences were small across platforms and were within the expected range of sampling error (95 % CI). In the Union Target File, WGS had the highest sensitivity (96.8 % SNVs; 95.0 % InDels), with ACE and SS sensitivities equal (94.9 % SNVs; 92.5 % InDels) in these GC-rich regions. Substantially reduced sensitivities (60-65 % SNVs; 48-58 % InDels) were observed with NG across all intervals. This was consistent with the steep reductions in coverage performance observed with NG among regions with GC fractions >50 % (Fig. 4).

## Discussion

The comprehensive nature of WGS and WES-based technologies means that most previous analytic performance studies have been independent of any particular disease or clinical scenario. In contrast, this study highlights issues of coverage and accuracy in a set of genes likely to be clinically relevant and provides a method of improving sensitivity in these regions. We demonstrate that several recently developed (2012–2014) commercial exome sequencing platforms continue to have significant gaps in their coverage of medically relevant genes. These deficiencies led us to design target regions, capture probes, and sequencing parameters that would improve both coverage and accuracy within these regions. An ACE strategy that ‘fills in’ gaps to a sufficient coverage depth for clinical interpretation and that expands coverage to more comprehensively cover medically interpretable areas of the genome, results in coverage efficiencies greater than other currently available platforms. Compared to conventional WES and 31× WGS, ACE shows a greater percentage of bases covered in the MIG (Figs. 2, 3, Additional file 4), the set of recently established ACMG secondary finding genes (Additional file 6), and variants known to be associated with disease (Fig. 5, Additional files 7 and 8) at coverage levels that are clinically relevant (≥20×).

**Fig. 6:**
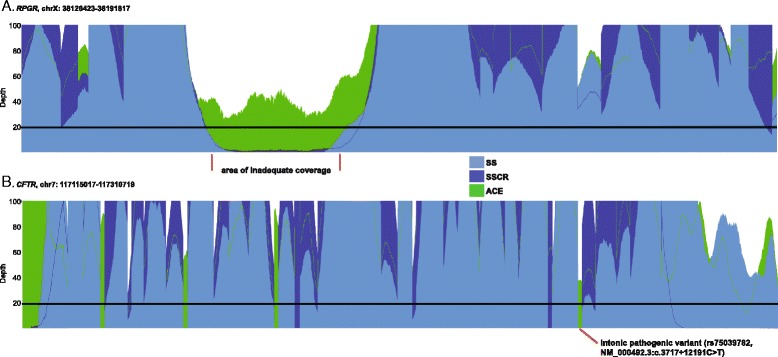
Coverage gaps in Retinitis Pigmentosa and Cystic Fibrosis genes are recovered with augmented exome approaches. Chromosomal position (x-axis) is plotted against coverage depth (y-axis) averaged over multiple 1000 Genome samples, with the clinical coverage threshold (≥20×) represented by a horizontal black line. Blue areas represent mean-depth of coverage across coding and non-coding regions using the SS (light blue), and SSCR (dark blue) exomes. Areas in green represent coverage gaps ‘filled in’ by ACE. These include areas with known pathogenic variants in high GC rich areas in the *RPGR* gene associated with retinitis pigmentosa (**a**); or non-coding regions of the *CFTR* gene (**b**)

The occurrence of ‘coverage gaps’ with conventional exome sequencing and their subsequent targeting by ACE is illustrated in *RPGR,* a gene in which over 300 mutations are implicated in retinitis pigmentosa; and *CFTR*, a gene in which >1,000 mutations are associated with cystic fibrosis. Figure 6 depicts the breadth and average depth of coverage in these genes, where coverage shortfalls are evident in areas where conventional exomes (blue) did not reach ≥20×. Targeting the sequence features described above, ACE ‘fills in’ missing coverage data so that the entire coding region and any clinically interpretable non-coding regions are covered at ≥20× (green). This includes a high GC content area in *RPGR* and an intronic region in *CFTR*. In the NA12878 sample, the percent of coding bases covered ranged from 71-87 % for *RPGR* at ≥20× using WES. One hundred percent and 88 % of coding bases were covered in *RPGR* at ≥20× using ACE and 31× WGS, respectively. Although conventional WES platforms captured 90-99 % of exonic bases at ≥20× in *CFTR,* an intronic pathogenic variant (rs75039782, NM_000492.3: c.3717 + 12191C > T) recommended for carrier screening [40] was only adequately covered using 31× WGS and ACE.

Increased coverage efficiency translated to improved accuracy when assessing observed variant calls against the reference calls-sets, with notable exceptions. In terms of sensitivity, ACE outperformed other platforms across all intervals (Table 1) whereas NX and NG had a substantially larger FN rate than other platforms, including WGS. Despite high coverage efficiency and finishing statistics (Figs. 2 and 3) relative to other WES platforms, NX showed relatively poor performance in terms of accuracy. This was a surprising result since we presumed that increased coverage efficiency would correlate directly with increased variant calling accuracy when all other parameters are fixed, including mean coverage depth. Interpreting the TP rates across various intervals (Table 1), it is likely that the lower sensitivities with NX and NG are due to a combination of inadequate coverage depth across what is predominantly coding regions (MIG, Common Target File) and relatively poor coverage outside of coding regions. Like SSCR, both NX and NG do not specifically target non-coding or regulatory regions, so poor performance in an interval that includes these regions is not unexpected. Unlike other platforms, however, this limitation in NG and NX had a substantial effect on the detection of variants that have moderate-high predicted functional impact (Union Target File).

Across intervals our results demonstrate that increased error rates occur in areas that are not sufficiently targeted by WES, due to either insufficient coverage of medically important regions or exclusion of non-coding regions of the genome. Whereas ACE and SS sensitivities are improved due to the specific expansion of coverage into UTRs, further improvements with ACE occur due to improved coverage in GC-rich regions (Table 2) and the selective inclusion of genomic regions (for example, areas near genes, promoter proximal sequences, splice recognition sequences) that are relevant for clinical interpretation despite their non-coding status. Targeting of ACE based on interpretability, emphasizing evidence of disease association and pathogenicity, results in higher coverage (Fig. 5, Additional files 7 and 8) and sensitivities (Table 1) to variants associated with disease or variants that are more likely to have deleterious effects.

Notably, there are limitations when drawing parallels between coverage and accuracy among platforms: (1) the NA12878 sample used in this study have variants occurring in only a small fraction of the regions being assessed; (2) areas that are medically relevant but may be challenging to sequence or that are error-prone (for example, low-complexity regions, regions of excessive coverage depth) are excluded from the current versions of the reference call-sets by design; (3) while reference call-sets serve as useful benchmarks, 16 % (12,558 / 78,489 regions) of the MIG and 12 % of known daSNVs in the 56 ACMG genes did not overlap loci in the GIBv2.18 high-confidence call-set; and (4) recent studies [25, 26] have shown that there is not a 1:1 correspondence between increases in coverage and increases in sensitivity . For a given loci, an increase in coverage from 10-20× would roughly translate to a 4-5 % increase in SNV sensitivity assuming an expected heterozygous/homozygous ratio of approximately 5/1 in an individual. This effect would be hard to detect using the reference call-sets in this study, as they represent a biased set of consistently covered loci compared to the unselected/unfiltered set of loci on which the coverage plots are based (Figs. 2, 3, and 4).

These limitations make it difficult to comprehensively resolve accuracy differences among platforms, despite obvious coverage differences in these areas (Figs. 2, 3, and 5, Additional files 4 and 5). Ongoing development [27, 41] of reference call-sets that leverage phased pedigree consistent variant calls and multiple reference materials may help increase the number of high-confidence variant calls in these regions. As these reference call-sets become more comprehensive, we anticipate that many additional variant observations will occur in GC-rich and known pathogenic regions of the genome that are specifically targeted by ACE but are not currently captured in sensitivity calculations due to reference set bias. As an example, we expanded out the canonical reference call-set to re-include high-quality calls that may have failed multi-dataset arbitration rules (that is, GIBv2.18 less restrictive call-set). By examining GC-rich areas of the genome across platforms with this reference call-set, we were able to reveal increases in sensitivity in the MIG with ACE (Table 2), although the numbers are relatively small.

A related concern, involves the interpretation of the FDR. Whereas TPs in the reference call-set are likely to be TPs given that they are called by multiple orthogonal technologies and pipelines, using the inverse of this set to confidently identify areas of the genome that are truly non-variant may not be justified. Recent evidence has shown that alignment-based [42] and some assembly-based [43] variant-callers show high error rates for large InDels and heterozygous InDels even at WGS coverage depths up to 90×. Although higher coverage (190×) WGS datasets contribute calls to the GiBv2.18 reference, the majority of datasets are <80×. In addition to difficulties in distinguishing InDels from other complex variants, larger variants and homopolymer runs in our sequenced datasets, the higher FDR for InDels across platforms (compared to SNVs) may reflect increased genotyping errors in the reference call-sets.

Alternative variant types, like structural variants, and alternative mechanisms of causal variation, like mosaicism, are not specifically evaluated in this study. Although methods to detect duplication and deletion events by exome-based sequencing methods continue to improve [44–46], they remain challenging to assess systematically on a genome-wide scale. Given the large fraction of disease heritability they are thought to represent [47], a reference call-set to enable accuracy comparisons among different platforms is needed. Improved reference datasets are being developed by NIST and others and will enable more objective comparisons between WES and WGS platforms for copy number variations. Similarly, the detection of mosaic variants in Mendelian disease is increasingly recognized as a clinically important and common mechanism of causal variation. Several recent studies using high-depth targeting sequencing approaches like gene panels [48, 49] and WES [7, 49–53] have shown the presence of somatic mutations capable of causing inherited disease when present in as little as 10 % of a patient’s cells. However, obtaining ≥20× local coverage depth on alternative alleles, when the fraction of cells in which the allele is present may be as low as 10 %, is not attainable with clinical WGS and conventional WES sequencing in a cost-effective manner. Conversely, the use of high coverage (>500×) gene panels increases the ability to resolve mosaic variants but only if they occur in the set of genes defined *a priori* in the panel - a limitation when attempting to diagnose a patient with atypical clinical manifestation or in the presence of substantial genetic heterogeneity [50]. For cases of inherited disorders and cancer, an ACE strategy that insures the availability of higher localized coverage depth and completeness of coverage within a comprehensive medically relevant target region is currently being assessed for its ability to resolve mosaic variants at low allele frequencies.

## Conclusions

The variation in coverage and accuracy among platforms highlights the need for clinicians to consider analytical performance when making clinical assessments, given the risk of over-interpreting negative results. At comparable levels of sequence data, ACE was the most sensitive enrichment-based platform among those tested; and was comparable to WGS despite an eight-fold reduction in the amount of sequence data obtained.

Considering that sequencing costs typically account for the largest fraction of total costs incurred when using exome-based assays in the clinic, this sensitivity makes ACE cost-efficient compared to conventional WES. This also makes ACE a cost-effective diagnostic tool compared to WGS given that WGS costs four to five times that of conventional WES for a given level of sensitivity based on sequencing costs alone [26]. In clinical applications such as inherited disease and tumor analysis where comprehensive coverage of medically interpretable areas of the genome requires higher localized sequencing depth, ACE offers both cost and performance advantages over other sequencing-based tests.

## Additional files

Additional file 1:
**Description of ACE assay construction and assessment of analytical validity (with data tables appended).** (PDF 126 kb) Additional file 2:
**ACE target file, defining regions of the genome targeted by the ACE assay used in this study.** Chromosomal position information is based on mapping to GRCh37. (XLSX 5275 kb) Additional file 3:
**Defines regions of the MIG using chromosomal position information and relevant gene identifiers; the reference transcript used for gene definitions; and the sources from which the genes were drawn.** (XLSX 4964 kb) Additional file 4:
**Re-analysis of coverage in the MIG and daSNV loci using WES/ACE data re-normalized to 12 Gb total sequence data.** (PDF 1215 kb) Additional file 5:
**Summary of NA12878 sequencing statistics across platforms, normalizing by total sequence amount or mean target coverage.** (PDF 63 kb) Additional file 6:
**Mean coverage and finishing statistics for 56 genes in the ACMG secondary findings list, using WES/ACE data normalized to both 12 Gb and 100× mean coverage.** (PDF 101 kb) Additional file 7:
**Percentage of disease associated SNV variant (daSNV) loci covered at >10×, >15×, >20×, and >25× local coverage depths by platform, using WES/ACE data normalized to both 12 Gb and 100× mean coverage.** (PDF 123 kb) Additional file 8:
**daSNVs in ACMG genes where inadequate coverage was observed among at least one platform, using WES/ACE data normalized to both 12 Gb and 100× mean coverage.** (XLSX 312 kb) Additional file 9:
**Common Target File representing regions common in ACE, SS, SSCR, NX, NG (that is, the intersection of platform specific target files), mapped to GRCh37.** (XLSX 2666 kb) Additional file 10:
**Union Target File representing regions aggregated across ACE, SS, SSCR, NX, NG (that is, the union of platform specific target files) that contain loci associated with moderate and high-impact variants, mapped to GRCh37.** (XLSX 181 kb) Additional file 11:
**Catalogue of exonic regions observed with >70 % high GC content.** (XLSX 15115 kb) Additional file 12:
**Summary of resources used in constructing reference regions and evaluating accuracy.** (PDF 42 kb)

## Abbreviations

ACE: Accuracy and Content Enhanced Augmented Exome
ACMG: American College of Medical Genetics
CEPH: Consanguinity in Centre d’Étude du Polymorphisme Humain
COSMIC: Cancer Gene Census
CPIC: Clinical Pharmacogenetics Implementation Consortium
FP: False Positive
FN: False Negative
GATK: Genome Analysis Toolkit
Gb: giga base pairs
GIB: Genome in a Bottle
GTR: Genetic Testing Registry
HD: High Depth
HGMD: Human Gene Mutation Database
HGNC: Human Gene Nomenclature Committee
ICCG: International Collaboration for Clinical Genomics
InDel: Insertion/Deletion
LC: Low Complexity
MIG: Medically Interpretable Genome
NG: NimbleGen SeqCap EZ Human Exome Library v3.0
NGS: Next Generation Sequencing
NIST: National Institutes of Standards and Technology
NX: llumina’s Nextera Rapid Exome Enrichment
OMIM: Online Mendelian Inheritance in Man
daSNV: Disease associated SNV
RM: Reference Material
SNV: Single Nucleotide Variant
SRA: Sequence Read Archive
SS: Agilent SureSelect Human All Exon v5 plus untranslated regions (UTR)
SSCR: Agilent SureSelect Clinical Research Exome
TP: True Positive
VQSR: Variant Quality Score Recalibration
VCF: Variant Call Format
VIP: Very Important Pharmacogenes
WES: Whole Exome Sequencing
WGS: Whole Genome Sequencing
