# Supplementary material for: Achieving high-sensitivity for clinical applications using augmented exome sequencing
Source: Genome Med. 2015 Jul 16;7(1):71. doi: 10.1186/s13073-015-0197-4 (PMC4534066; doi:10.1186/s13073-015-0197-4)
Supplement: Additional file 1: — Description of ACE assay construction and assessment of analytical validity (with data tables appended). (PDF 126 kb) [file 13073_2015_197_MOESM1_ESM.pdf]

Additional File 1: Description of ACE, analytical assessment and target region

Based on previous studies of discordant variant calls among multiple sequencing platforms[1, 2] and further work in elucidating the mechanisms underlying these errors, a database of problematic genomic regions was constructed[3]. Problematic regions are those in which high variability in coverage and increased (>3-fold) variant calling error rates were consistently observed across platforms, samples and sequencing algorithms. While empirically low coverage or mismapping in these regions may be due to a variety of sequence features (e.g. redundant paralogous sequences, repetitive content, breakpoint library regions, areas of repeat collapse), a large fraction of these problematic regions (~45%) are comprised of 100bp regions having >70% GC content.

We developed a proprietary sample preparation, enrichment, and sequencing protocol to supplement targeting in high-GC content regions occurring in 8,020 biomedically and medically relevant genes. We defined the target set of 8,020 genes using established causal gene-phenotype relationships documented in primary literature and sets of genes with potential clinical impact. Accordingly, we optimized targeting and sequencing in genes 1) associated with Mendelian/monogenic disease in a clinical or research setting; 2) implicated in a pharmacological drug response; and 3) implicated in a biological process or regulatory pathway closely linked to a disease process (e.g. in cancer). Genes were chosen based on evidence in public data sources (International Collaboration for Clinical Genomics, Human Gene Mutation Database[4], Online Mendelian Inheritance in Man [5], ClinVar [6]) and supplemented with over 3,000 genes from our own curation of peer-reviewed publications. Manually curated publications were screened and selected based on the study design, publication date and the reported strength of association (effect size, expected pathogenicity) of the gene or variant. We included genes that may not yet have sufficient evidence for being causally implicated in disease or reached the level of evidence necessary for clinical reporting[7], with the expectation that many will be implicated in disease in the future. ACE includes regions covered in conventional exomes while 1) supplementing coverage in these genes and 2) extending coverage to non-coding regions of the exome that are biologically interpretable (e.g. UTRs, intergenic, intronic).

Following an enhanced capture protocol, captured target DNA was pooled with target DNA captured from standard enrichment (using the Agilent SureSelect Clinical Research Exome kit) prior to sequencing. A target file, defining regions of the genome targeted by the ACE assay used in this study is included in **Additional_file2.bed.** The ACE assay results shown in this study were performed as part of the ACE Clinical Exome, a standardized diagnostic test available through the Personalis CLIA and CAP accredited laboratory.

The assay results in the main body of this paper were downsampled to control the total amount of sequence data or mean coverage in order to enable comparisons among multiple WES platforms at fixed levels. In contrast, ACE Clinical Exome runs typically vary in the total number of reads produced and the mean coverage obtained in the target region. The accuracy and precision results below are based on runs that demonstrate this expected variation.

Analytical accuracy and positive percent agreement (PPA) across replicates

Eight replicates of the same sample (NA12878) were run multiple times. Six replicates were run within a single instrument using two operators; with two additional replicates run among two additional instruments over two additional days. For each replicate the whole process started from the same reference DNA tubes. Each replicate varied in the total GB amount, mean coverage and number of finished genes as shown below:

Replicate ID	IAC411A1	BAR352A5	IAC410A1	BAR352A10	BAR351A10	BAR352A11	BAR351A5	BAR351A11
Gb	14.7	11.8	12.4	13.1	13.4	15.2	16.5	18.4
mean coverage	104.6	83.4	88.9	96.8	100.9	109.8	116.8	141.0
genes finished	7092	6606	6491	6892	6980	7034	7113	7186

operator	1	2	1	2	1	2	1	1
instrument	3	1	2	1	1	1	1	1

For each replicate, Personalis evaluated the accuracy of variant detection using publically available reference materials (RM) and reference datasets. Variants detected in the highly characterized Hapmap sample, NA12878 are compared with genotype calls in the National Institutes of Standards and Technology (NIST) NA12878 reference call-set (GIBv2.18[1]). NA12878 is also the first pilot NIST reference material sample. This GIBv2.18 call-set combines variant calls among multiple participating clinical and research laboratories, arbitrating calls among multiple platforms, sequencing protocols, and alignment/variant calling algorithms. GIBv2.18 contains a set of high-confidence variant and homozygous reference genotype calls.

The eight NA12878 samples were prepared, sequenced and analyzed by the Personalis genome sequencing pipeline, while varying both the operator and instrument. SNV and INDEL variants were stored in a VCF file and compared to GIBv2.18 within a region defined by the ACE target region.

We used the following methods in order to determine accuracy:

- Analytical sensitivity is based on the enumeration of true positives (TP) and false negatives (FN) and is calculated as:

  TP / (TP + FN)

- Analytical specificity is based on the enumeration of true negatives (TN) and false positives (FP) and is calculated as:

  TN / (TN +FP)

Due to the skewed class distribution between TN and TP rates (i.e. the number of TN is several magnitudes larger than TP) we also calculate the FDR. We used the following methods in order to determine analytic FDR:

- FDR is based on the enumeration of true positives (TP) and false positives (FP) and is calculated as:

  FP / (FP +TP)

TP are determined by the number of calls that are variant in the observed variant call-set and have a matching genotype in the GIBv2.18 call-set. FN are determined by calls that are variant in the GIBv2.18 call-set and that have a missing or non-matching genotype in the observed variant call-set. TN are determined by the number of matching homozygous reference sites in both the GIBv2.18 and observed variant call-sets. FP are determined by the number of variant calls in the observed call-set that have a non-matching genotype in the GIBv2.18. 95% confidence intervals were based on an exact binomial test.

Based on comparison of the observed NA12878 variant calls with the GIBv2.18 call-set, the analytical sensitivity of ACE ranged between 98.8% - 99.1% (SNVs) and 88.7%-90.7% (InDels) within the ACE target region. The analytical specificity was determined to be >99.9% for both SNVs and InDels for all replicates. The FDR was 0.1% (SNVs) across replicates and ranged between and 5.9%-7.2% across replicates for InDels. Ranges are given across two operators and across three different instruments (**Table 1**).

Based on sensitivity estimates, Positive Percent Agreement (PPA) ranged from 96.9-98.7 among SNVs and 85.9-90.1 among InDels (**Table 2**). PPA was based on genotype matching within callable (QC-passed) variants occurring within the ACE target region.

Stability of assay over multiple runs varying operator and instrument

Twenty-three replicates drawn from seven samples were run, across three instruments and two operators. For each sample new libraries were prepared. Agreement and reproducibility measures were based on sequencing statistics thought to impact variant call accuracy, including:

1. Duplications Rate and Capture Specificity: Good quality libraries should have duplication rates of less than 20% since library diversity assures balanced allelic representation. Additionally, capture specificities of >40% are required to insure efficient exome enrichment. All sample runs had consistently <10% of PCR duplicates and >60% capture specificity in the exome target regions (**Table 3**).
2. Average Base Quality: An average base quality of >Q30.0 was required within both the ACE Clinical Exome target region for every sample run, corresponding to >99.9% confidence in the base calls. An average base quality of >Q33.0 was achieved within the ACE Clinical Exome target region for all assay runs (**Table3**).
3. Finishing: Personalis ACE Clinical Exome augments coverage in ~8,000 additional genes currently known to be associated with human disease. Within this subset, >6,000 genes are required to have >99.00% of their constitutive coding bases achieve ≥20x local coverage depth, a level of coverage depth necessary to call heterozygous SNVs with ~99% sensitivity in WES and WGS data[4]. A gene is termed “finished” when reaching this threshold. All replicate samples achieved >6,900 genes with >99.00% of the constitutive coding exons at ≥20x coverage. A consistent set of 6,129 genes (>99.00% at >20x) and 4,989 genes (100% at >20x) were finished among all runs (**Table 3**).

Variation across samples based on capture specificity, duplication rate, and average base quality was summarized by the coefficient of variation (CV). Within instrument and operators, CVs ranged from 0.8-1.1% (base quality); 1.0-2.2% (capture specificity); and 24.0-33.4% (duplication rate)**.** A studentized t-test indicated no significant ($p<0.05$) differences for capture specificity X operator ($p=.24$) and average base quality X operator ($p=.49$). Duplication rate and mean coverage was significantly ($p=.02$) higher among operator 2 measures. However, this did not translate into significant differences in the number of genes finished (**Table 4**).

Table 1: Accuracy across replicates

Sample (Coriell)	instrument	operator	Sample ID	TP	FP	%sensitivity (95% CI)*	%specificity (95% CI)*	%FDR
NA12878	1	1	BAR351A5	40488	22	99.1[99.0-99.2]	99.9%(>99.9%)	0.1[<0.1-0.1]
				1558	111	90.0[88.4-91.3]	99.9%(>99.9%)	6.7[5.5-8.0]
NA12878	1	1	BAR351A10	40699	25	99.0[98.9-99.1]	99.9%(>99.9%)	0.1[<0.1-0.1]
				1538	119	88.7[87.2-90.2]	99.9%(>99.9%)	7.2[6.0-8.5]
NA12878	1	1	BAR351A11	40882	37	99.1[99.1-99.2]	99.9%(>99.9%)	0.1[<0.1-0.1]
				1572	102	90.7[89.2-92.0]	99.9%(>99.9%)	6.1[5.0-7.3]
NA12878	1	2	BAR352A5	40754	33	98.8[98.7-98.9]	99.9%(>99.9%)	0.1[<0.1-0.1]
				1557	97	89.9[88.4-91.3]	99.9%(>99.9%)	5.9[4.8-7.1]
NA12878	1	2	BAR352A10	40482	29	98.8[98.7-98.9]	99.9%(>99.9%)	0.1[<0.1-0.1]
				1543	106	89.0[87.5-90.5]	99.9%(>99.9%)	6.4[5.3-7.7]
NA12878	1	2	BAR352A11	40663	37	99.1[99.0-99.2]	99.9%(>99.9%)	0.1[<0.1-0.1]
				1567	119	90.4[88.9-91.8]	99.9%(>99.9%)	7.1[5.9-8.4]
NA12878	2	1	IAC410A1	40848	32	98.9[98.8-99.0]	99.9%(>99.9%)	0.1[<0.1-0.1]
				1557	97	90.1[88.6-91.5]	99.9%(>99.9%)	5.9[4.8-7.1]
NA12878	3	1	IAC411A1	40568	40	99.1[99.0-99.1]	99.9%(>99.9%)	0.1[<0.1-0.1]
				1565	115	90.3[88.8-91.6]	99.9%(>99.9%)	6.8[5.7-8.2]

*comparisons are made with GIBv2.18 from Zook et. al.[8] for high-confident reference calls within an interval defined by ACE target region

Table 2: Positive Percent Agreement across operator and instruments

	IAC411A1	BAR352A5	IAC410A1	BAR352A10	BAR351A10	BAR352A11	BAR351A5	BAR351A11
Gb	14.7	11.8	12.4	13.1	13.4	15.2	16.5	18.4
mean coverage	104.6	83.4	88.9	96.8	100.9	109.8	116.8	141.0
genes finished (100% at 20x)	7092	6606	6491	6892	6980	7034	7113	7186
operator	1	2	1	2	1	2	1	1
instrument	3	1	2	1	1	1	1	1

**SNVs**	IAC411A1	BAR352A5	IAC410A1	BAR352A10	BAR351A10	BAR352A11	BAR351A5	BAR351A11
IAC411A1	NA	98.1	98.2	98.0	97.9	98.0	98.5	98.4
BAR352A5	-	NA	98.3	97.9	98.6	98.5	98.7	98.1
IAC410A1	-	-	NA	97.9	98.7	98.5	98.7	98.3
BAR352A10	-	-	-	NA	97.9	97.7	98.3	97.5
BAR351A10	-	-	-	-	NA	98.4	97.6	99.9
BAR352A11	-	-	-	-	-	NA	98.6	97.8
BAR351A5	-	-	-	-	-	-	NA	96.9
BAR351A11	-	-	-	-	-	-	-	NA

**InDel**	IAC411A1	BAR352A5	IAC410A1	BAR352A10	BAR351A10	BAR352A11	BAR351A5	BAR351A11
IAC411A1	NA	88.4	88.4	88.6	88.3	87.9	88.1	88.8
BAR352A5	-	NA	87.0	86.9	88.0	89.5	85.9	90.1
IAC410A1	-	-	NA	87.4	87.0	86.2	86.5	87.1
BAR352A10	-	-	-	NA	87.0	87.6	86.1	87.9
BAR351A10	-	-	-	-	NA	87.9	88.2	88.0
BAR352A11	-	-	-	-	-	NA	87.3	87.4
BAR351A5	-	-	-	-	-	-	NA	89.1
BAR351A11	-	-	-	-	-	-	-	NA

*PPA based on genotype (not just position) matching among callable variants within the ACE target region.

PPA = TP * 100 / (TP + FN)

Table 3: Sequencing statistics and gene finishing over multiple ACE runs

Sample (Coriell)	Sample ID	instrument	operator/day	sequence amt (Gb)	capture specificity	duplication rate	average base quality	mean coverage	genes finished (>99.00% at ≥20x)*	genes finished (100% at ≥20x)**
NA24149	BAR351A1	1	1/1	14.0	62.2%	4.3%	34.4	99.5	7309	6916
NA12878	BAR351A10	1	1/1	13.4	64.6%	2.3%	34.2	100.9	7338	6980
NA12878	BAR351A11	1	1/1	18.4	65.6%	2.2%	33.6	141.0	7439	7186
NA24143	BAR351A2	1	1/1	12.5	62.1%	4.2%	34.3	88.5	7145	6667
NA24385	BAR351A3	1	1/1	12.4	61.8%	4.3%	34.3	87.7	7178	6680
SEC1-01	BAR351A4	1	1/1	14.3	61.1%	4.2%	34.2	99.4	7316	6931
NA12878	BAR351A5	1	1/1	16.5	62.1%	4.6%	34.3	116.8	7396	7113
NA18799	BAR351A6	1	1/1	14.8	62.7%	4.5%	34.3	106.3	7313	6990
NA12444	BAR351A7	1	1/1	10.5	62.7%	4.4%	34.0	75.6	6947	6340
NA18799	BAR351A9	1	1/1	15.5	65.0%	2.5%	34.2	117.7	7426	7146
NA24149	BAR352A1	1	2/2	12.5	63.5%	6.4%	34.0	88.4	7211	6728
NA12878	BAR352A10	1	2/2	13.1	63.9%	3.3%	33.8	96.8	7293	6892
NA12878	BAR352A11	1	2/2	15.2	62.1%	2.5%	33.8	109.8	7367	7034
NA24143	BAR352A2	1	2/2	11.1	63.3%	6.3%	34.1	78.5	7012	6422
NA24385	BAR352A3	1	2/2	12.8	63.4%	6.4%	33.9	90.6	7246	6803
SEC1-01	BAR352A4	1	2/2	12.0	62.9%	6.2%	34.3	84.2	7128	6590
NA12878	BAR352A5	1	2/2	11.8	63.3%	6.1%	34.1	83.4	7142	6606
NA18799	BAR352A6	1	2/2	12.9	64.2%	6.7%	34.2	92.9	7248	6831
NA12444	BAR352A7	1	2/2	10.4	64.0%	7.0%	33.6	74.4	6936	6262
NA12444	BAR352A8	1	2/2	9.9	64.1%	3.4%	33.5	73.1	6916	6197
NA18799	BAR352A9	1	2/2	12.5	63.2%	3.3%	34.3	91.1	7246	6798
NA12878	IAC410A1	2	1/3	12.4	63.0%	3.9%	33.2	88.9	7008	6491
NA12878	IAC411A1	3	1/3	14.7	62.0%	3.5%	33.7	104.6	7393	7092

*assuming the average gene contains 10^4 base pairs, we estimate finishing at four decimal places (>.9900 or 99.00%). A consistent set of 6,129 genes (>99.00% at >20x) and 4,989 genes (100% at >20x) were finished among all 23 runs above.

Table 4: Run-Run variation by operator/day

Coefficient of Variation (CV)	Overall	within operator 1	within operator 2
capture specificity	1.8	2.2	1.0
duplication rate	34.1	24	32.4
base quality	0.9	1.1	0.8
mean coverage	16.8	16.9	12.1

	operator 1 mean	operator 2 mean	p-value
capture specificity	62.9	63.4	p=0.25
duplication rate	3.7	5.2	p=0.02
base quality	34.1	34.0	p=0.49
mean coverage	102.2	87.6	p=0.02
finished genes (>99.00% at >20x)	7267.3	7158.6	p=0.11
finished genes (100% at >20x)	6877.7	6651.2	p=0.06

References

1. Lam HYK, Clark MJ, Chen R, Chen R, Natsoulis G, O'Huallachain M, Dewey FE, Habegger L, Ashley EA, Gerstein MB, Butte AJ, Ji HP, Snyder M: **Performance comparison of whole-genome sequencing platforms**. *Nat Biotechnol* 2012, **30**:78–82.

2. Clark MJ, Chen R, Lam HYK, Karczewski KJ, Chen R, Euskirchen G, Butte AJ, Snyder M: **Performance comparison of exome DNA sequencing technologies**. *Nat Biotechnol* 2011, **29**:908–914.

3. Patwardhan A, Clark M, Morgan A, Chervitz S, Pratt M, Bartha G, Chandratillake G, Garcia S, Leng N, Chen R: **Variant priorization and analysis incorporating problematic regions of the genome**. *Pac Symp Biocomput Pac Symp Biocomput* 2014:277–287.

4. Stenson PD, Mort M, Ball EV, Shaw K, Phillips A, Cooper DN: **The Human Gene Mutation Database: building a comprehensive mutation repository for clinical and molecular genetics, diagnostic testing and personalized genomic medicine**. *Hum Genet* 2014, **133**:1–9.

5. Amberger J, Bocchini C, Hamosh A: **A new face and new challenges for Online Mendelian Inheritance in Man (OMIM®)**. *Hum Mutat* 2011, **32**:564–567.

6. Landrum MJ, Lee JM, Riley GR, Jang W, Rubinstein WS, Church DM, Maglott DR: **ClinVar: public archive of relationships among sequence variation and human phenotype**. *Nucleic Acids Res* 2014, **42**(Database issue):D980–985.

7. Richards CS, Bale S, Bellissimo DB, Das S, Grody WW, Hegde MR, Lyon E, Ward BE, Molecular Subcommittee of the ACMG Laboratory Quality Assurance Committee: **ACMG recommendations for standards for interpretation and reporting of sequence variations: Revisions 2007**. *Genet Med Off J Am Coll Med Genet* 2008, **10**:294–300.

8. Zook JM, Chapman B, Wang J, Mittelman D, Hofmann O, Hide W, Salit M: **Integrating human sequence data sets provides a resource of benchmark SNP and indel genotype calls**. *Nat Biotechnol* 2014, **32**:246–251.
