# Supplementary material for: Achieving high-sensitivity for clinical applications using augmented exome sequencing
Source: Genome Med. 2015 Jul 16;7(1):71. doi: 10.1186/s13073-015-0197-4 (PMC4534066; doi:10.1186/s13073-015-0197-4)
Supplement: Additional file 4: — Re-analysis of coverage in the MIG and daSNV loci using WES/ACE data re-normalized to 12 Gb total sequence data. (PDF 1215 kb) [file 13073_2015_197_MOESM4_ESM.pdf]

Additional File 4: Reanalysis of coverage in the MIG and in daSNV loci using WES/ACE data re-normalized to 12Gb of total sequence data.

Shown below are images from a re-analysis of coverage in the MIG, GC-rich regions, and daSNVs after re-normalizing WES/ACE data to a total of 12Gb of sequence data. Relative performance between platforms and the differences observed between platforms were consistent with Figures 2-5 presented in the main body of the paper, which were based on WES/ACE normalized to a mean coverage level of 100x in the respective target regions.

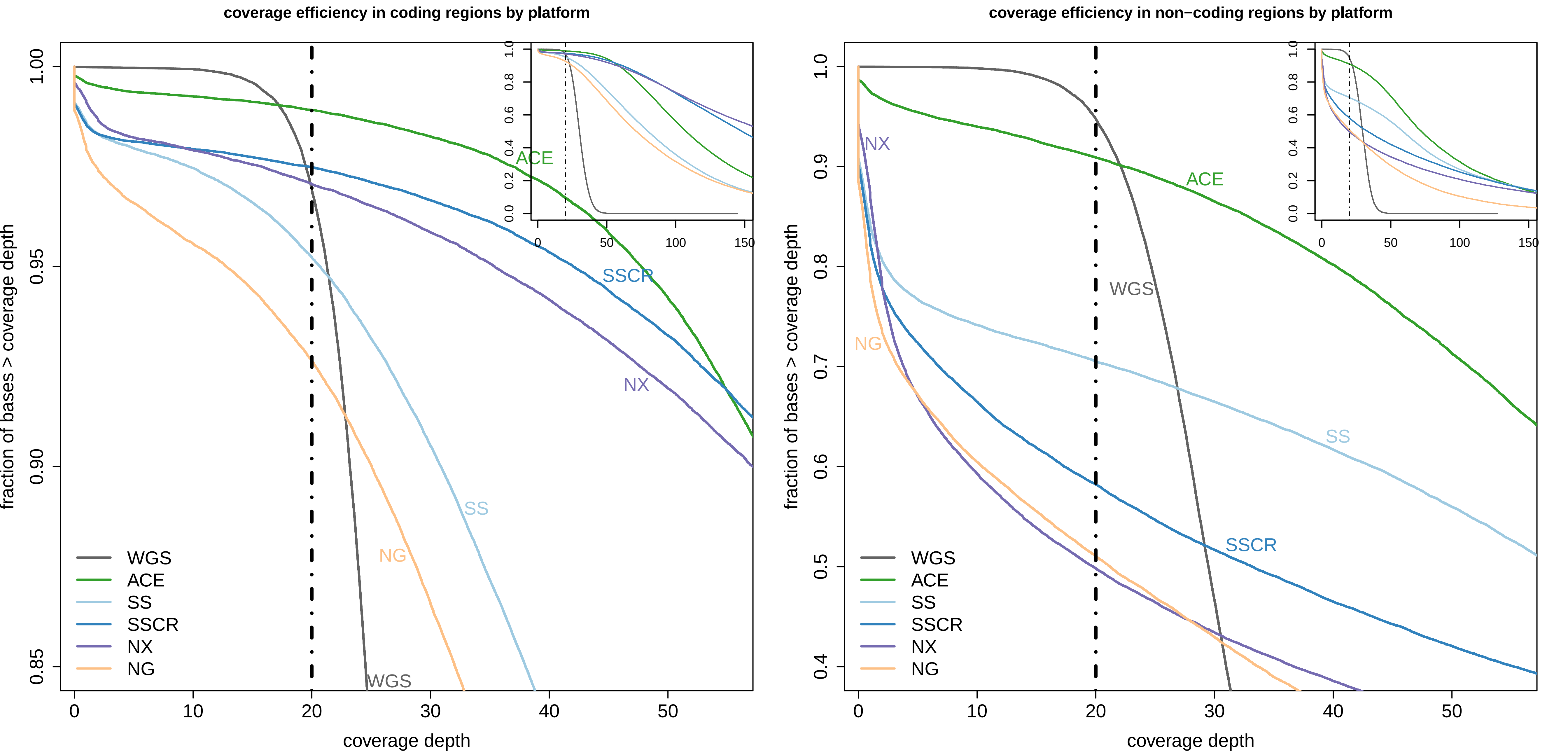

coverage efficiency in coding regions by platform
fraction of bases > coverage depth
coverage depth
WGS
ACE
SS
SSCR
NX
NG
coverage efficiency in non−coding regions by platform
fraction of bases > coverage depth
coverage depth
WGS
ACE
SS
SSCR
NX
NG

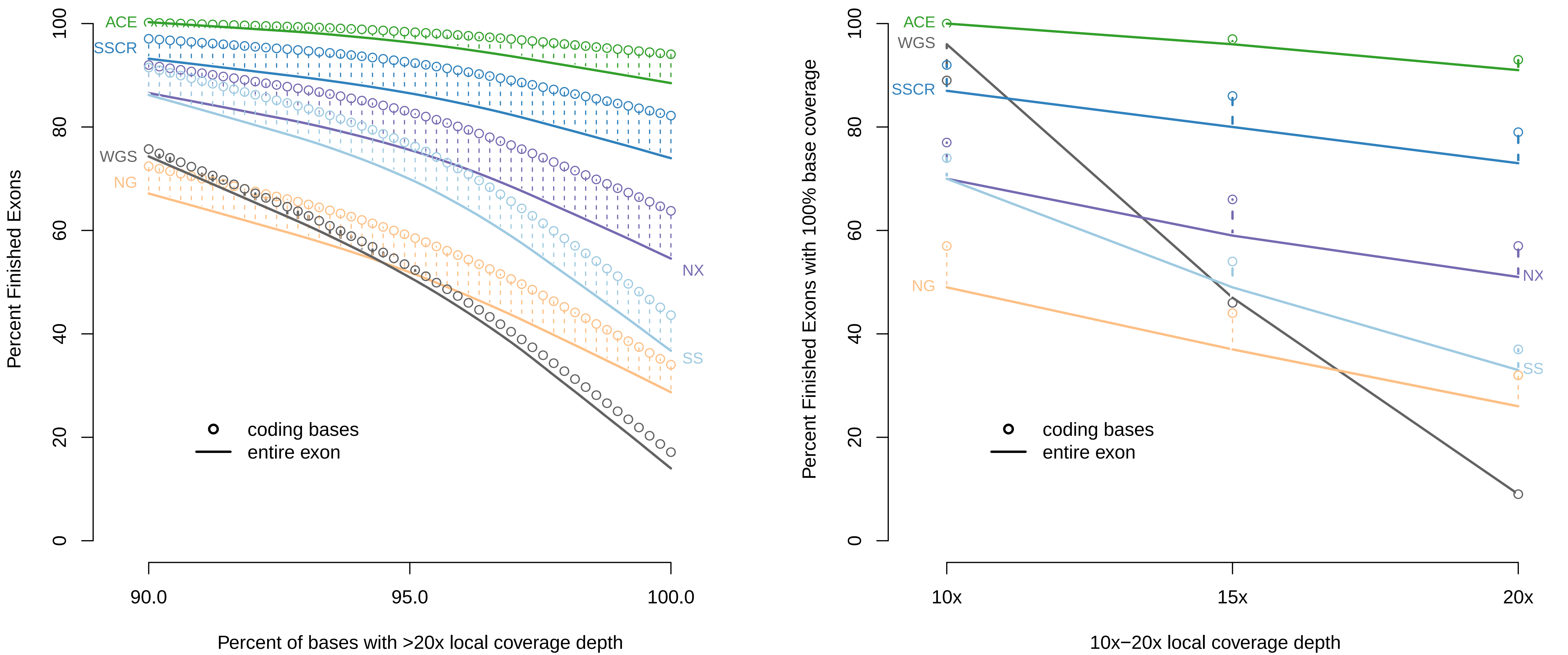

Percent Finished Exons
0
20
40
60
80
100
ACE
SSCR
WGS
NG
NX
SS
coding bases
entire exon
90.0
95.0
100.0
Percent of bases with >20x local coverage depth
Percent Finished Exons with 100% base coverage
ACE
WGS
SSCR
NG
NX
SS
coding bases
entire exon
10x
15x
20x
10x−20x local coverage depth

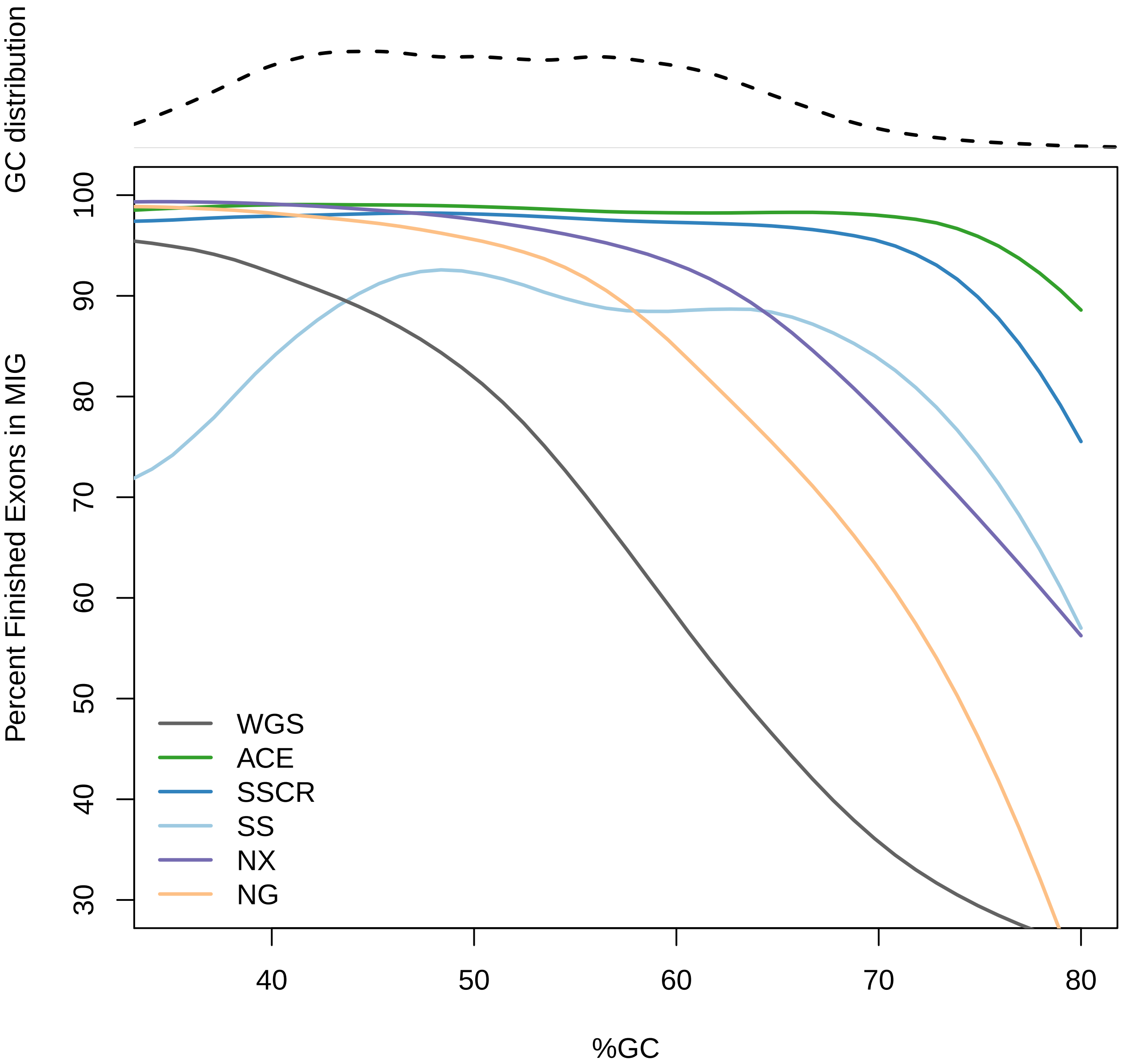

GC distribution
Percent Finished Exons in MIG
100
90
80
70
60
50
40
30
WGS
ACE
SSCR
SS
NX
NG
40
50
60
70
80
%GC

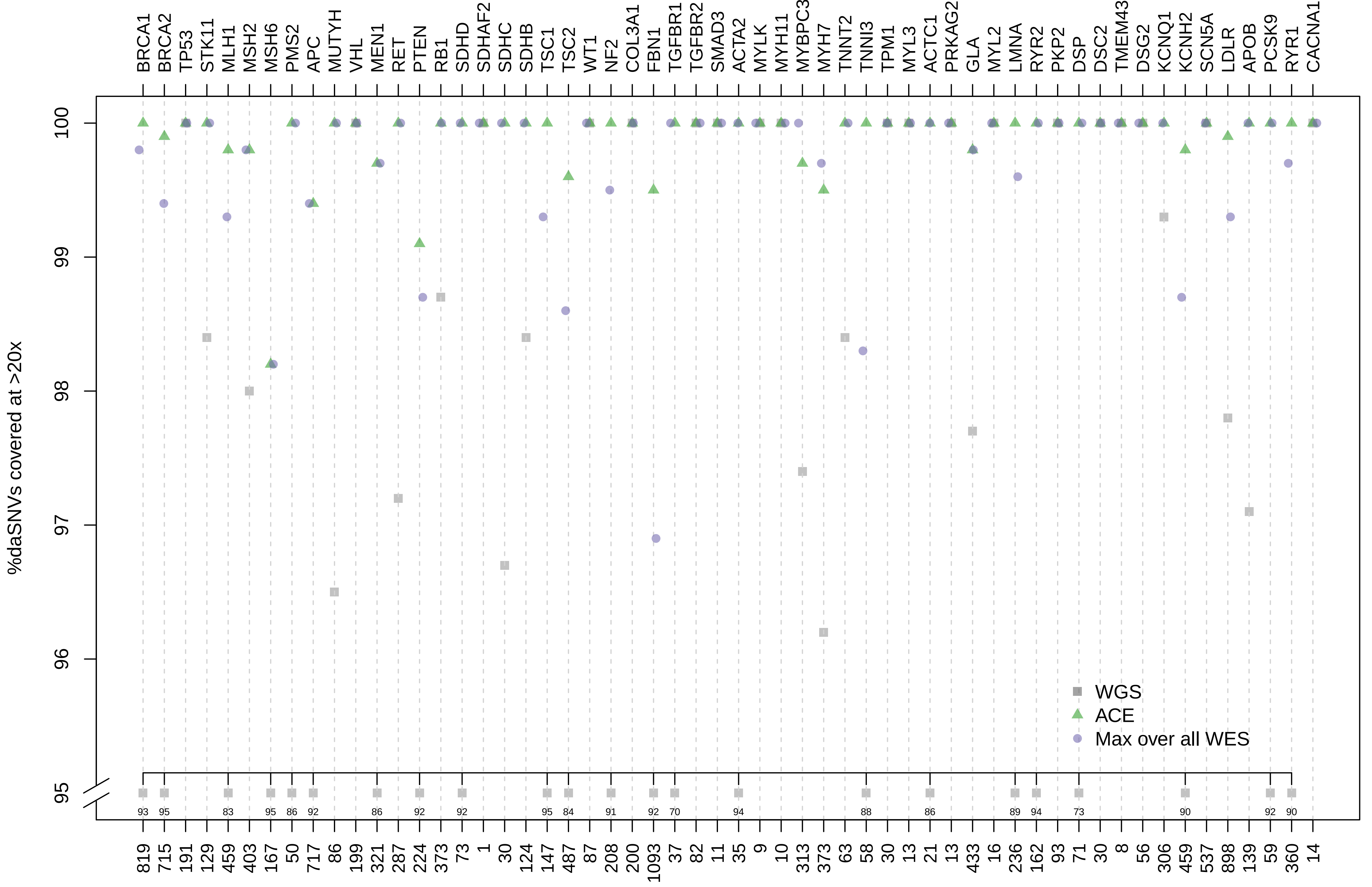
BRCA1 BRCA2 TP53 STK11 MLH1 MSH2 MSH6 PMS2 APC MUTYH VHL MEN1 RET PTEN RB1 SDHD SDHAF2 SDHC SDHB TSC1 TSC2 WT1 NF2 COL3A1 FBN1 TGFBR1 TGFBR2 SMAD3 ACTA2 MYLK MYH11 MYBPC3 MYH7 TNNT2 TNNI3 TPM1 MYL3 ACTC1 PRKAG2 GLA MYL2 LMNA RYR2 PKP2 DSP DSC2 TMEM43 DSG2 KCNQ1 KCNH2 SCN5A LDLR APOB PCSK9 RYR1 CACNA1
%daSNVs covered at >20x
100
99
98
97
96
95
WGS
ACE
Max over all WES
93 95 83 95 86 92 86 92 92 95 84 91 92 70 94 88 86 89 94 73 90 92 90
819 715 191 129 459 403 167 50 717 86 199 321 287 224 373 73 1 30 124 147 487 87 208 200 1093 37 82 11 35 9 10 313 373 63 58 30 13 21 13 433 16 236 162 93 71 30 8 56 306 459 537 898 139 59 360 14
