# Supplementary material for: Achieving high-sensitivity for clinical applications using augmented exome sequencing
Source: Genome Med. 2015 Jul 16;7(1):71. doi: 10.1186/s13073-015-0197-4 (PMC4534066; doi:10.1186/s13073-015-0197-4)
Supplement: Additional file 5: — Summary of NA12878 sequencing statistics across platforms, normalizing by total sequence amount or mean target coverage. (PDF 63 kb) [file 13073_2015_197_MOESM5_ESM.pdf]

Additional file 5. Summary of NA12878 sequencing statistics across platforms, normalizing by total sequence amount or mean target coverage. MIG= medically interpretable genome; CS = Capture Specificity; Seq = Sequence; Dup = Duplication Rate

									Mean coverage				Mean coverage	
Platform	Content (Mb)	Baits	Insert Size	Seq Length	CS	Base Quality	Seq (Gb)*	Dup (%)	target	MIG	Seq (Gb)	Dup (%)	target*	MIG
WGS	--	--	312.5 +/-64.8	101	--	32.6	100	--	31.5**	29.5	100	--	31.5**	29.5
ACE	69	RNA	220.4 +/-56.4	100	62.1	33.8	12	2.0	86.9	120.3	13.8	2.3	100x	138.0
SS	75	RNA	212.5 +/-51.0	100	73.4	33.0	12	7.7	88.3	90.4	13.8	8.7	100x	102.7
SSCR	54	RNA	210.3 +/-50.7	100	80.6	33.4	12	8.6	132.2	164.4	8.9	5.5	100x	125.1
NX	37	DNA	243.9 +/-81.1	35-150	55.3	31.7	12	8.3	91.1	199.4	18.6	10.5	100x	208.8
NG	64	DNA	235.3 +/-34.4	100	73.5	31.2	12	5.4	91.9	86.9	13.4	7.0	100x	95.5

*WES/ACE normalized based on amount of total sequence data or mean coverage in the platform-specific target region
**For WGS, coverage is shown across the whole genome in lieu of a target region
