# Supplementary material for: Achieving high-sensitivity for clinical applications using augmented exome sequencing
Source: Genome Med. 2015 Jul 16;7(1):71. doi: 10.1186/s13073-015-0197-4 (PMC4534066; doi:10.1186/s13073-015-0197-4)
Supplement: Additional file 6: — Mean coverage and finishing statistics for 56 genes in the ACMG secondary findings list, using WES/ACE data normalized to both 12 Gb and 100× mean coverage. (PDF 101 kb) [file 13073_2015_197_MOESM6_ESM.pdf]

Additional file 6. Mean coverage and finishing statistics for 56 genes in the ACMG secondary findings list. Each cell indicates the gene-specific mean coverage observed (top number) and the percentage of exon bases covered at ≥20x (bottom number) by platform. The second row summarizes the number of genes with 100.0% of exonic bases covered at ≥20x. WES and ACE data normalized by total sequence amount (left) or mean target coverage (right).

	**WES/ACE Normalize to 12Gb**						**WES/ACE Normalize to 100x**					
HGNC Symbol (HGNC ID)	SS	SSCR	NX	NG	WGS	ACE	SS	SSCR	NX	NG	WGS	ACE
genes with 100.0% bases covered at≥20x	**13**	**42**	**40**	**6**	**2**	**51**	**15**	**39**	**36**	**12**	**2**	**51**
BRCA1 (1100.0)	176.4; 100.0	295.7; 100.0	370.7; 100.0	126.1; 99.6	34.6; 100.0	185.6; 100.0	201.8; 100.0	218.9; 100.0	371.1; 100.0	137.7; 100.0	34.6; 100.0	215.2; 100.0
BRCA2 (1101)	86.6; 99.1	184.4; 100.0	214.1; 100.0	90.2; 99.7	35.0; 99.7	115.9; 100.0	98.4; 99.4	140.4; 100.0	223.9; 100.0	100.5; 100.0	35.0; 99.7	134.2; 100.0
TP53 (11998)	96.9; 94.1	149.2; 100.0	236.9; 100.0	49.6; 86.0	25.1; 90.4	120.4; 100.0	110.7; 95.5	110.8; 100.0	249.2; 100.0	56.3; 88.0	25.1; 90.4	140.8; 100.0
STK11 (11389)	79.5; 100.0	118.3; 100.0	110.1; 98.8	41.7; 80.7	28.6; 95.9	87.2; 100.0	91.7; 100.0	86.8; 100.0	119.2; 99.9	48.2; 85.4	28.6; 95.9	100.4; 100.0
MLH1 (7127)	126.5; 100.0	204.8; 100.0	316.9; 100.0	120.2; 99.9	30.3; 98.0	139.1; 100.0	143.4; 100.0	153.9; 100.0	331.5; 100.0	130.9; 99.9	30.3; 98.0	159.0; 100.0
MSH2 (7325)	68.9; 99.0	222.2; 100.0	300.2; 100.0	120.7; 98.0	31.2; 94.5	151.3; 100.0	77.0; 100.0	165.5; 100.0	310.3; 100.0	131.9; 99.0	31.2; 94.5	172.1; 100.0
MSH6 (7329)	83.0; 61.4	147.6; 100.0	269.2; 99.7	58.7; 95.5	35.2; 99.2	105.9; 100.0	94.0; 97.7	112.4; 100.0	278.8; 99.8	66.0; 98.3	35.2; 99.2	118.5; 100.0
PMS2 (9122)	80.0; 76.5	224.6; 99.7	288.5; 100.0	244.3; 97.6	25.4; 82.5	146.9; 96.0	91.1; 78.6	168.9; 96.4	294.3; 99.9	253.8; 98.3	25.4; 82.5	169.3; 99.0
APC (583)	112.9; 99.2	228.6; 100.0	329.3; 100.0	63.0; 95.5	31.2; 97.9	144.9; 100.0	127.4; 99.2	172.8; 100.0	340.9; 100.0	72.4; 97.8	31.2; 97.9	168.6; 100.0
MUTYH (7527)	109.1; 99.6	140.7; 100.0	251.6; 100.0	56.6; 95.0	26.5; 86.5	99.1; 100.0	126.6; 99.9	104.9; 100.0	258.4; 100.0	62.3; 98.3	26.5; 86.5	112.8; 100.0
VHL (12687)	105.6; 88.9	156.7; 100.0	279.9; 100.0	59.0; 47.0	26.5; 79.9	112.6; 100.0	121.9; 92.1	112.7; 92.5	289.2; 100.0	64.4; 56.5	26.5; 79.9	131.6; 100.0
MEN1 (7010)	77.6; 76.7	105.1; 77.5	125.0; 77.5	40.9; 61.0	27.2; 94.0	80.9; 91.3	88.2; 76.8	83.6; 77.5	131.1; 77.5	47.0; 66.2	27.2; 94.0	92.3; 91.4
RET (9967)	101.4; 94.2	167.0; 100.0	222.6; 100.0	52.4; 89.8	26.4; 92.2	152.5; 100.0	116.0; 95.6	125.0; 100.0	234.9; 100.0	58.1; 93.7	26.4; 92.2	173.9; 100.0
PTEN (9588)	104.1; 100.0	255.2; 100.0	305.8; 100.0	151.5; 100.0	35.9; 100.0	164.9; 100.0	121.7; 100.0	190.4; 100.0	312.9; 100.0	173.9; 100.0	35.9; 100.0	187.7; 100.0
RB1 (9884)	64.1; 93.4	182.9; 98.0	276.4; 100.0	116.5; 95.1	33.3; 93.4	134.3; 100.0	72.3; 95.5	136.3; 96.7	282.4; 99.7	128.0; 95.1	33.3; 93.4	153.2; 100.0
SDHD (10683)	63.0; 65.4	135.5; 94.7	329.2; 100.0	174.6; 100.0	31.0; 98.2	103.5; 100.0	72.0; 65.4	101.4; 94.1	329.1; 100.0	195.5; 100.0	31.0; 98.2	116.2; 100.0
SDHAF2 (26034)	154.2; 100.0	275.6; 100.0	375.2; 100.0	65.1; 99.6	32.0; 99.4	170.7; 100.0	170.1; 100.0	203.1; 100.0	378.8; 100.0	68.6; 99.8	32.0; 99.4	203.7; 100.0
SDHC (10682)	69.3; 94.0	161.6; 100.0	315.6; 100.0	130.9; 100.0	29.9; 97.8	101.1; 100.0	76.0; 97.8	119.7; 100.0	316.7; 100.0	146.3; 100.0	29.9; 97.8	112.6; 100.0
SDHB (10681)	84.7; 99.4	172.6; 100.0	357.7; 100.0	85.9; 100.0	31.9; 96.0	121.2; 100.0	95.4; 100.0	133.5; 100.0	373.2; 100.0	96.2; 100.0	31.9; 96.0	139.7; 100.0

TSC1 (12362)	89.5; 91.9	218.9; 100.0	279.6; 100.0	69.1; 99.6	30.0; 96.8	144.2; 100.0	103.8; 93.5	166.6; 100.0	288.2; 100.0	74.9; 99.6	30.0; 96.8	165.2; 100.0
TSC2 (12363)	87.0; 95.4	152.5; 100.0	223.2; 100.0	58.4; 94.9	26.6; 88.2	159.7; 100.0	99.5; 96.2	116.3; 100.0	233.8; 100.0	66.7; 96.9	26.6; 88.2	181.6; 100.0
WT1 (12796)	60.9; 99.8	120.9; 99.0	208.4; 99.9	56.4; 68.6	25.9; 88.3	133.3; 100.0	70.7; 100.0	92.3; 98.4	217.7; 97.5	67.1; 69.8	25.9; 88.3	151.1; 100.0
NF2 (7773)	72.7; 98.8	175.2; 100.0	413.3; 100.0	82.6; 89.9	28.8; 95.9	130.1; 100.0	82.7; 100.0	133.9; 100.0	418.7; 100.0	86.2; 90.1	28.8; 95.9	148.0; 100.0
COL3A1 (2201)	59.4; 88.4	159.7; 100.0	258.7; 100.0	96.0; 100.0	31.4; 97.1	100.0; 100.0	66.5; 92.1	118.4; 99.8	266.4; 100.0	103.4; 100.0	31.4; 97.1	115.1; 100.0
FBN1 (3603)	95.3; 98.4	218.7; 100.0	336.7; 100.0	79.5; 97.5	30.6; 97.2	147.9; 100.0	107.7; 99.2	166.3; 100.0	353.9; 100.0	86.9; 98.0	30.6; 97.2	168.6; 100.0
TGFBR1 (11772)	110.7; 93.6	303.0; 93.6	331.6; 93.6	106.7; 93.6	30.6; 94.3	202.0; 100.0	123.9; 93.6	229.4; 93.6	332.8; 93.6	115.7; 93.6	30.6; 94.3	234.4; 100.0
TGFBR2 (11773)	110.4; 100.0	216.1; 100.0	270.4; 99.2	57.0; 79.1	29.4; 98.0	151.2; 100.0	125.3; 100.0	165.7; 100.0	277.9; 98.6	64.3; 77.5	29.4; 98.0	175.3; 100.0
SMAD3 (6769)	83.4; 95.9	150.5; 100.0	237.2; 100.0	39.3; 83.9	26.5; 97.1	142.8; 100.0	95.6; 99.4	114.5; 100.0	256.2; 100.0	41.2; 89.8	26.5; 97.1	160.3; 100.0
ACTA2 (130)	78.6; 98.5	197.6; 100.0	238.4; 100.0	73.3; 99.9	29.5; 94.2	155.2; 100.0	88.2; 98.5	145.1; 100.0	252.2; 100.0	84.4; 100.0	29.5; 94.2	175.4; 100.0
MYLK (7590)	101.7; 99.1	209.1; 100.0	254.4; 98.5	70.2; 94.6	28.2; 94.7	141.1; 100.0	115.9; 99.2	155.5; 100.0	267.1; 98.5	75.5; 97.1	28.2; 94.7	162.3; 100.0
MYH11 (7569)	119.0; 99.6	191.9; 100.0	328.8; 100.0	91.4; 98.8	26.6; 92.8	129.6; 100.0	135.9; 99.9	146.9; 100.0	339.5; 100.0	100.5; 98.5	26.6; 92.8	147.9; 100.0
MYBPC3 (7551)	95.9; 97.6	149.9; 100.0	54.3; 83.6	52.3; 87.7	26.0; 85.8	146.0; 100.0	108.7; 98.3	110.3; 100.0	60.9; 84.8	56.7; 91.0	26.0; 85.8	166.3; 100.0
MYH7 (7577)	78.3; 90.5	180.4; 98.7	310.2; 100.0	79.8; 97.3	26.6; 90.3	134.6; 100.0	88.1; 92.5	135.7; 97.9	326.6; 100.0	88.5; 97.9	26.6; 90.3	155.5; 100.0
TNNT2 (11949)	97.8; 100.0	156.7; 100.0	342.0; 100.0	77.9; 99.0	29.3; 98.8	121.6; 100.0	114.0; 100.0	119.8; 100.0	353.6; 100.0	85.6; 99.4	29.3; 98.8	141.6; 100.0
TNNI3 (11947)	73.2; 96.2	156.5; 100.0	209.8; 100.0	61.9; 91.0	26.1; 92.3	158.9; 100.0	85.9; 98.0	116.8; 100.0	225.3; 100.0	75.2; 100.0	26.1; 92.3	183.7; 100.0
TPM1 (12010)	141.3; 100.0	197.5; 100.0	297.5; 100.0	67.3; 80.9	28.6; 96.8	133.4; 100.0	158.7; 100.0	153.1; 100.0	300.9; 100.0	76.1; 80.9	28.6; 96.8	151.8; 100.0
MYL3 (7584)	76.6; 100.0	171.1; 100.0	216.7; 100.0	59.8; 100.0	28.4; 94.0	118.7; 100.0	87.8; 100.0	129.2; 100.0	231.0; 100.0	70.6; 100.0	28.4; 94.0	140.1; 100.0
ACTC1 (129)	82.5; 100.0	181.2; 100.0	172.8; 100.0	62.9; 99.8	28.5; 94.1	157.9; 100.0	94.8; 100.0	137.1; 100.0	178.7; 99.9	70.8; 100.0	28.5; 94.1	180.1; 100.0
PRKAG2 (9386)	92.1; 98.5	177.1; 100.0	248.2; 100.0	74.3; 93.8	29.1; 94.5	124.9; 100.0	104.9; 99.0	136.3; 96.3	262.1; 100.0	81.7; 96.3	29.1; 94.5	142.9; 100.0
GLA (4296)	73.3; 96.0	189.1; 100.0	312.9; 100.0	82.5; 97.8	28.8; 96.0	131.9; 100.0	84.6; 99.3	145.2; 100.0	323.0; 100.0	91.7; 100.0	28.8; 96.0	150.9; 100.0
MYL2 (7590)	111.0; 100.0	175.1; 100.0	333.4; 100.0	64.5; 96.8	30.4; 95.0	130.4; 100.0	125.9; 100.0	137.2; 100.0	341.8; 100.0	69.2; 96.8	30.4; 95.0	145.9; 100.0
LMNA (6636)	66.3; 88.2	107.7; 99.3	203.1; 100.0	39.1; 70.0	26.2; 90.4	89.9; 100.0	77.6; 89.7	82.7; 97.5	215.0; 100.0	44.1; 74.4	26.2; 90.4	103.9; 100.0
RYR2 (10484)	81.9; 98.8	197.0; 100.0	164.0; 99.2	112; 98.0	30.3; 96.0	124.8; 100.0	93.3; 99.2	151.5; 100.0	172.3; 99.3	122.2; 98.6	30.3; 96.0	142.5; 100.0
PKP2 (9024)	53.8; 81.9	164.4; 96.1	241.2; 98.3	89.1; 85.9	27.7; 92.0	108.3; 100.0	61.2; 88.8	125.9; 93.4	251.9; 98.6	95.3; 87.1	27.7; 92.0	125; 100.0
DSP (3052)	98.3; 99.2	201.2; 100.0	315.6; 100.0	53.7; 85.6	30.5; 98.2	125.8; 100.0	110.8; 99.5	154.6; 100.0	326.7; 100.0	58.0; 81.5	30.5; 98.2	145.8; 100.0
DSC2 (3036)	95.3; 93.8	213.4; 100.0	328.8; 100.0	111.9; 97.5	31.4; 98.5	152.0; 100.0	111.0; 96.3	164; 100.0	338.3; 100.0	123.1; 97.5	31.4; 98.5	173.6; 100.0

TMEM43 (28472)	84.6; 100.0	131.5; 100.0	197.0; 100.0	87.1; 93.0	27.4; 98.8	101.5; 100.0	97.7; 99.9	102.5; 100.0	207.5; 100.0	98.5; 97.8	27.4; 98.8	114.8; 100.0
DSG2 (3049)	89.6; 98.7	196.2; 100.0	337.3; 97.1	73.0; 97.2	32.5; 97.0	127.9; 100.0	100.9; 99.0	148.9; 100.0	345.2; 97.2	78.9; 97.4	32.5; 97.0	145.6; 100.0
KCNQ1 (6294)	82.8; 91.5	120.7; 92.3	172.8; 87.6	41.0; 70.4	24.1; 77.7	122.4; 99.3	95.8; 93.3	93.3; 91.5	184.2; 95.7	47.1; 76.3	24.1; 77.7	141.4; 99.6
KCNH2 (6251)	65.9; 91.5	138.9; 97.6	113.3; 92.2	42.6; 68.1	24.9; 86.4	106.4; 100.0	75.5; 92.4	106.6; 97.5	117.5; 93.5	47.8; 70.1	24.9; 86.4	121.9; 100.0
SCN5A (10593)	83.5; 98.6	184.3; 98.7	141.9; 93.3	68.2; 97.7	26.0; 92.3	142.3; 98.7	95.8; 98.7	140.8; 98.7	149.5; 93.7	77.1; 97.5	26.0; 92.3	162.6; 98.7
LDLR (6547)	101.2; 100.0	202.1; 100.0	255.8; 100.0	65.7; 97.7	26.8; 86.7	154.2; 100.0	114.2; 99.8	152.6; 100.0	271.7; 100.0	74.7; 99.8	26.8; 86.7	177.5; 100.0
APOB (603)	108.5; 98.5	211.0; 100.0	286.9; 100.0	107.0; 99.1	31.1; 97.4	133.1; 100.0	123.0; 99.4	161.1; 100.0	299.0; 99.7	116.7; 99.2	31.1; 97.4	152.2; 100.0
PCSK9 (20001)	58.0; 88.5	107.0; 99.8	169.1; 96.8	60.4; 95.2	26.7; 91.2	91.2; 100.0	65.9; 88.8	81.7; 99.6	186.4; 97.2	68.9; 95.8	26.7; 91.2	105.1; 100.0
RYR1 (10483)	77.5; 91.1	143.4; 98.4	243.4; 98.3	45.9; 84.2	26.2; 87.8	111.2; 99.9	88.9; 92.1	108.5; 97.9	256.7; 98.4	50.4; 86.1	26.2; 87.8	127.4; 100.0
CACNA1S (1397)	82.1; 99.7	159.2; 100.0	249.8; 100.0	54.9; 95.7	27.9; 93.3	123.8; 100.0	92.9; 99.7	120.6; 100.0	261.8; 100.0	60.9; 96.4	27.9; 93.3	141.2; 100.0
