# Supplementary material for: Achieving high-sensitivity for clinical applications using augmented exome sequencing
Source: Genome Med. 2015 Jul 16;7(1):71. doi: 10.1186/s13073-015-0197-4 (PMC4534066; doi:10.1186/s13073-015-0197-4)
Supplement: Additional file 7: — Percentage of disease associated SNV variant (daSNV) loci covered at >10×, >15×, >20×, and >25× local coverage depths by platform, using WES/ACE data normalized to both 12 Gb and 100× mean coverage. (PDF 123 kb) [file 13073_2015_197_MOESM7_ESM.pdf]

Additional file 7. Percentage of disease associated SNV variant (daSNV) loci covered at ≥10x, ≥15x, ≥20x and ≥25x local coverage depths by platform. For each gene, the total number of daSNVs identified is shown in the second column. For each gene and minimum coverage level, the highest observed percentage of daSNVs covered among all conventional exome platforms (SS, SSCR, NX, NG) was selected and reported as Max. The percentage of loci covered with ACE/WGS is shown separately. Results are shown for both WES/ACE data normalized to 12Gb total sequence data (left) or 100x mean coverage (right).

		WES/ACE data normalized to 12Gb total sequence data												WES/ACE data normalized to 100x mean target coverage											
		≥10x			≥15x			≥20x			≥25x			≥10x			≥15x			≥20x			≥25x		
Gene (HGNC ID)	daSNV	Max[a]	WGS	ACE	Max	WGS	ACE	Max	WGS	ACE	Max	WGS	ACE	Max[a]	WGS	ACE	Max	WGS	ACE	Max	WGS	ACE	Max	WGS	ACE
BRCA1 (1100)	819	100	100	100	100	98.9	100	99.8	93.4	100	99.8	72.4	100	100	100	100	100	98.9	100	99.8	93.4	100	98.9	72.3	100
BRCA2 (1101)	715	99.4	100	99.9	99.4	99.6	99.9	99.4	95	99.9	99.4	74.5	99.7	99.4	100	99.9	99.4	99.6	99.9	99.4	94.8	99.9	99.4	74.5	99.9
TP53 (11998)	191	100	100	100	100	100	100	100	100	100	100	96.3	100	100	100	100	100	100	100	100	100	100	100	96.3	100
STK11 (11389)	129	100	100	100	100	100	100	100	98.4	100	100	79.1	100	100	100	100	100	100	100	100	98.4	100	100	79.1	100
MLH1 (7127)	459	99.6	100	99.8	99.6	100	99.8	99.3	83.2	99.8	99.3	43.8	99.6	99.6	100	99.8	99.6	100	99.8	99.3	83.2	99.8	99.3	43.8	99.8
MSH2 (7325)	403	99.8	100	99.8	99.8	100	99.8	99.8	98	99.8	99.8	90.3	99.8	99.8	100	99.8	99.8	100	99.8	99.8	98	99.8	99.8	90.3	99.8
MSH6 (7329)	167	98.2	100	98.2	98.2	100	98.2	98.2	94.6	98.2	98.2	78.4	98.2	98.2	100	98.2	98.2	100	98.2	98.2	94.6	98.2	98.2	78.4	98.2
PMS2 (9122)	50	100	100	100	100	100	100	100	86	100	100	56	100	100	100	100	100	100	100	100	86	100	100	56	100
APC (583)	717	99.4	100	99.4	99.4	98.5	99.4	99.4	92.5	99.4	99.4	73.9	99.4	99.4	100	99.4	99.4	98.5	99.4	99.4	92.5	99.4	99.2	73.9	99.4
MUTYH (7527)	86	100	100	100	100	98.8	100	100	96.5	100	100	73.3	100	100	100	100	100	98.8	100	100	96.5	100	100	73.3	100
VHL (12687)	199	100	100	100	100	100	100	100	100	100	100	97.5	100	100	100	100	100	100	100	100	100	100	100	97.5	100
MEN1 (7010)	321	99.7	100	99.7	99.7	97.5	99.7	99.7	86.3	99.7	99.7	65.1	99.7	99.7	100	99.7	99.7	97.5	99.7	99.7	86.3	99.7	99.7	65.1	99.7
RET (9967)	287	100	100	100	100	100	100	100	97.2	100	100	88.5	100	100	100	100	100	100	100	100	97.2	100	100	88.5	100
PTEN (9588)	224	99.1	100	99.6	99.1	100	99.6	98.7	92	99.1	98.2	77.7	99.1	99.1	100	99.6	99.1	100	99.6	99.1	91.5	99.6	98.2	77.7	99.1
RB1 (9884)	373	100	100	100	100	100	100	100	98.7	100	100	79.9	100	100	100	100	100	100	100	100	98.7	100	100	79.9	100
SDHD (10683)	73	100	100	100	100	100	100	100	91.8	100	100	72.6	100	100	100	100	100	100	100	100	91.8	100	100	72.6	100
SDHAF2 (26034)	1	100	100	100	100	100	100	100	100	100	100	100	100	100	100	100	100	100	100	100	100	100	100	100	100
SDHC (10682)	30	100	100	100	100	100	100	100	96.7	100	100	93.3	100	100	100	100	100	100	100	100	96.7	100	100	93.3	100

SDHB (10681)	124	100	100	100	100	100	100	100	98.4	100	100	87.9	100	100	100	100	100	100	100	100	98.4	100	100	87.9	100
TSC1 (12362)	147	100	100	100	100	100	100	99.3	94.6	100	99.3	75.5	100	100	100	100	100	100	100	100	94.6	100	99.3	75.5	100
TSC2 (12363)	487	99.6	99.6	100	99.2	96.5	99.6	98.6	83.8	99.6	98.4	61	99.6	99.6	99.6	100	99.6	96.5	100	99.4	83.8	99.6	97.7	61	99.6
WT1 (12796)	87	100	100	100	100	100	100	100	100	100	100	83.9	100	100	100	100	100	100	100	100	100	100	100	83.9	100
NF2 (7773)	208	99.5	100	100	99.5	99.5	100	99.5	91.3	100	99.5	71.6	100	99.5	100	100	99.5	99.5	100	99.5	91.3	100	99.5	71.6	100
COL3A1 (2201)	200	100	100	100	100	100	100	100	100	100	100	91.5	100	100	100	100	100	100	100	100	100	100	100	91.5	100
FBN1 (3603)	1093	97.3	100	99.7	97.1	99	99.5	96.9	91.5	99.5	96.9	69.8	99.4	98.4	100	99.7	97.2	99	99.5	96.9	91.5	99.5	96.9	69.8	99.5
TGFBR1 (11772)	37	100	100	100	100	100	100	100	70.3	100	100	59.5	100	100	100	100	100	100	100	100	70.3	100	100	59.5	100
TGFBR2 (11773)	82	100	100	100	100	100	100	100	100	100	100	98.8	100	100	100	100	100	100	100	100	100	100	100	98.8	100
SMAD3 (6769)	11	100	100	100	100	100	100	100	100	100	100	100	100	100	100	100	100	100	100	100	100	100	100	100	100
ACTA2 (130)	35	100	100	100	100	100	100	100	94.3	100	100	65.7	100	100	100	100	100	100	100	100	94.3	100	100	65.7	100
MYLK (7590)	9	100	100	100	100	100	100	100	100	100	100	100	100	100	100	100	100	100	100	100	100	100	100	100	100
MYH11 (7569)	10	100	100	100	100	100	100	100	100	100	100	70	100	100	100	100	100	100	100	100	100	100	100	70	100
MYBPC3 (7551)	313	100	100	100	100	100	100	100	97.4	99.7	100	79.6	99.7	100	100	100	100	100	100	100	97.4	99.7	100	79.6	99.7
MYH7 (7577)	373	100	99.2	100	99.7	98.7	100	99.7	96.2	99.5	99.7	76.7	99.5	99.7	99.2	100	99.5	98.7	100	99.5	96.2	99.7	99.5	76.7	99.5
TNNT2 (11949)	63	100	100	100	100	100	100	100	98.4	100	100	85.7	100	100	100	100	100	100	100	100	98.4	100	100	85.7	100
TNNI3 (11947)	58	98.3	100	100	98.3	100	100	98.3	87.9	100	98.3	67.2	100	98.3	100	100	98.3	100	100	98.3	87.9	100	98.3	67.2	100
TPM1 (12010)	30	100	100	100	100	100	100	100	100	100	100	100	100	100	100	100	100	100	100	100	100	100	100	100	100
MYL3 (7584)	13	100	100	100	100	100	100	100	100	100	100	100	100	100	100	100	100	100	100	100	100	100	100	100	100
ACTC1 (129)	21	100	100	100	100	100	100	100	85.7	100	100	47.6	100	100	100	100	100	100	100	100	85.7	100	100	47.6	100
PRKAG2 (9386)	13	100	100	100	100	100	100	100	100	100	100	84.6	100	100	100	100	100	100	100	100	100	100	100	84.6	100
GLA (4296)	433	99.8	100	99.8	99.8	100	99.8	99.8	97.7	99.8	99.8	71.4	99.8	99.8	100	99.8	99.8	100	99.8	99.8	97.7	99.8	99.8	71.4	99.8
MYL2 (7590)	16	100	100	100	100	100	100	100	100	100	100	50	100	100	100	100	100	100	100	100	100	100	100	50	100
LMNA (6636)	236	100	100	100	100	98.7	100	99.6	88.6	100	99.6	62.7	100	100	100	100	100	98.7	100	100	88.6	100	99.6	63.1	100
RYR2 (10484)	162	100	100	100	100	99.4	100	100	93.8	100	100	67.3	100	100	100	100	100	99.4	100	100	93.8	100	100	67.3	100
PKP2 (9024)	93	100	100	100	100	100	100	100	100	100	100	86	100	100	100	100	100	100	100	100	100	100	100	86	100
DSP (3052)	71	100	100	100	100	100	100	100	73.2	100	100	50.7	100	100	100	100	100	100	100	100	73.2	100	100	50.7	100
DSC2 (3036)	30	100	100	100	100	100	100	100	100	100	100	90	100	100	100	100	100	100	100	100	100	100	100	90	100
TMEM43 (28472)	8	100	100	100	100	100	100	100	100	100	100	100	100	100	100	100	100	100	100	100	100	100	100	100	100

DSG2 (3049)	56	100	100	100	100	100	100	100	100	100	100	100	100	100	100	100	100	100	100	100	100	100	100	100	100
KCNQ1 (6294)	306	100	100	100	100	100	100	100	99.3	100	100	90.5	100	100	100	100	100	100	100	100	99.3	100	100	90.5	100
KCNH2 (6251)	459	99.1	99.8	99.8	98.9	99.1	99.8	98.7	90.2	99.8	98.3	63.4	99.1	98.9	99.8	100	98.9	99.1	99.8	98.7	90.4	99.8	98	63.4	99.6
SCN5A (10593)	537	100	100	100	100	100	100	100	100	100	100	98.5	100	100	100	100	100	100	100	100	100	100	100	98.5	100
LDLR (6547)	898	99.8	100	100	99.6	99.7	99.9	99.3	97.8	99.9	99.1	94.4	99.9	99.8	100	100	99.3	99.7	99.9	98.8	97.8	99.9	98.7	94.4	99.9
APOB (603)	139	100	100	100	100	100	100	100	97.1	100	100	83.5	100	100	100	100	100	100	100	100	97.1	100	100	83.5	100
PCSK9 (20001)	59	100	100	100	100	100	100	100	91.5	100	100	52.5	100	100	100	100	100	100	100	100	91.5	100	100	52.5	100
RYR1 (10483)	360	99.7	100	100	99.7	97.8	100	99.7	89.7	100	99.7	68.1	99.7	99.7	100	100	99.7	97.8	100	99.7	89.7	100	99.7	68.1	99.7
CACNA1S (1397)	14	100	100	100	100	100	100	100	100	100	100	92.9	100	100	100	100	100	100	100	100	100	100	100	92.9	100

[a]For brevity, we report only the highest obtained percentage achieved (Max) across all conventional WES platforms.
