# Supplementary material for: Achieving high-sensitivity for clinical applications using augmented exome sequencing
Source: Genome Med. 2015 Jul 16;7(1):71. doi: 10.1186/s13073-015-0197-4 (PMC4534066; doi:10.1186/s13073-015-0197-4)
Supplement: Additional file 12: — Summary of resources used in constructing reference regions and evaluating accuracy. (PDF 42 kb) [file 13073_2015_197_MOESM12_ESM.pdf]

Additional file 12. Summary of resources used in constructing reference regions and evaluating accuracy.

Resource	Location	Purpose
NISTIntegratedCalls_14datasets_131103_allcall_UGHapMerge_HetHomVarPASS_VQSRv2.18_all_nouncert_excludesimplerep_excludesegdups_excludedecoy_excludeRepSeqSTRs_noCNVs.vcf.gz	ftp://ftp-trace.ncbi.nih.gov/giab/ftp/data/NA12878/variant_calls/NIST/	constructing GIBv2.18 high-confident call-set
union13callableMQonlymerged_addcert_nouncert_excludesimplerep_excludesegdups_excludedecoy_excludeRepSeqSTRs_noCNVs_v2.18_2mindatasets_5minYesNoRatio.bed.gz	ftp://ftp-trace.ncbi.nih.gov/giab/ftp/data/NA12878/variant_calls/NIST/	constructing GIBv2.18 high-confident call-set
NISTIntegratedCalls_14datasets_131103_allcall_UGHapMerge_HetHomVarPASS_VQSRv2.18_all.primitives.vcf.gz	ftp://ftp-trace.ncbi.nih.gov/giab/ftp/data/NA12878/variant_calls/NIST/	constructing GIBv2.18 less-restrictive call-set
union13callableMQonlymerged_addcert_nouncert_v2.18_2mindatasets_5minYesNoRatio.bed.gz	ftp://ftp-trace.ncbi.nih.gov/giab/ftp/data/NA12878/variant_calls/NIST/	constructing GIBv2.18 less-restrictive call-set
Additional_file3.xlsx	Included in supplemental spreadsheets	Regions and source databases defining the MIG. Locations based on NCBI annotation (release 105). Used in evaluating coverage and accuracy in medically relevant regions.
Additional_file9.xlsx	Included in supplemental spreadsheets	target regions overlapping in ACE, SS, SSCR, NX, NG target files (i.e. the intersection of all WES/ACE specific target regions). These regions are combined with calls in the high-confidence call-set or less-restrictive call-sets to evaluate accuracy among platforms
Additional_file10.txt	Included in supplemental spreadsheets	target regions aggregated across WES/ACE target files (i.e. the union of all WES/ACE specific target files). These regions are combined with calls in the high-confidence call-set or less-restrictive call-sets to evaluate accuracy among platforms
Additional_file11.xlsx	Included in supplemental spreadsheets	Regions in which the GC fraction was observed to be >70% using a 100bp moving window in NA12878. Combined with calls in the less-restrictive call-set to evaluate accuracy in high-GC regions
Platform-specific FASTQ files and .vcf files	SRA PRJNA289286 and available upon request	Sequence data and observed SNV and InDel variant calls
